# HMGB1 promotes mitochondrial transfer between hepatocellular carcinoma cells through RHOT1 and RAC1 under hypoxia

**DOI:** 10.1038/s41419-024-06536-6

**Published:** 2024-02-20

**Authors:** Mengjia Jing, Xiaofeng Xiong, Xin Mao, Qianben Song, Lumiao Zhang, Yiming Ouyang, Yingzhi Pang, Yu Fu, Wei Yan

**Affiliations:** 1 Department of Gastroenterology, Tongji Hospital, Tongji Medical College, Huazhong University of Science and Technology, Wuhan, 430030 China; 2 Department of Gastroenterology, Union Hospital, Tongji Medical College, Huazhong University of Science and Technology, Wuhan, 430022 China

**Keywords:** Metastasis, Cancer microenvironment

## Abstract

Mitochondrial transfer plays an important role in various diseases, and many mitochondrial biological functions can be regulated by HMGB1. To explore the role of mitochondrial transfer in hepatocellular carcinoma (HCC) and its relationship with HMGB1, field emission scanning electron microscopy, immunofluorescence, and flow cytometry were used to detect the mitochondrial transfer between HCC cells. We found that mitochondrial transfer between HCC cells was confirmed using tunnel nanotubes (TNTs). The transfer of mitochondria from the highly invasive HCC cells to the less invasive HCC cells could enhance the migration and invasion ability of the latter. The hypoxic conditions increased the mitochondrial transfer between HCC cells. Then the mechanism was identified using co-immunoprecipitation, luciferase reporter assay, and chromatin immunoprecipitation. We found that RHOT1, a mitochondrial transport protein, promoted mitochondrial transfer and the migration and metastasis of HCC cells during this process. Under hypoxia, HMGB1 further regulated RHOT1 expression by increasing the expression of NFYA and NFYC subunits of the NF-Y complex. RAC1, a protein associated with TNTs formation, promoted mitochondrial transfer and HCC development. Besides, HMGB1 regulated RAC1 aggregation to the cell membrane under hypoxia. Finally, the changes and significance of related molecules in clinical samples of HCC were analyzed using bioinformatics and tissue microarray analyses. We found that HCC patients with high HMGB1, RHOT1, or RAC1 expression exhibited a relatively shorter overall survival period. In conclusion, under hypoxic conditions, HMGB1 promoted mitochondrial transfer and migration and invasion of HCC cells by increasing the expression of mitochondrial transport protein RHOT1 and TNTs formation-related protein RAC1.

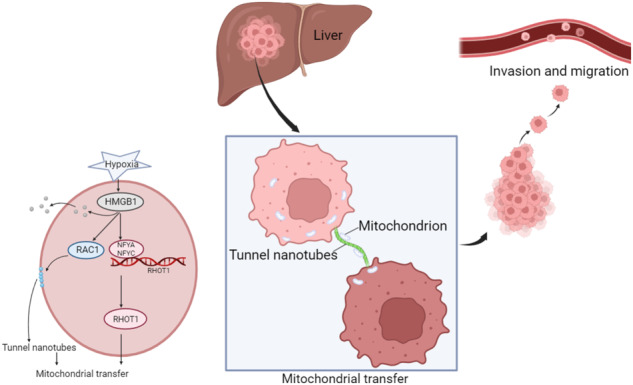

## Introduction

Hepatocellular carcinoma (HCC) is one of the most common and fatal cancer-related causes of death worldwide [1]. Early metastasis is the primary reason for the poor prognosis of HCC, and the first step for HCC to metastasize is that cancer cells gain invasiveness [2]. Numerous studies have shown that changes in tumor microenvironment, mitochondria, and other cellular substructures play an important role in tumor progression [3–5]. Elucidating these changes and the mechanisms underlying HCC progression may provide a new perspective for exploring treatment strategies for HCC.

As a dynamic regulator of all malignant cell processes, mitochondrial plasticity represents the potential of tumor cells in tumor transformation, development and progression, metastasis and diffusion, and therapeutic response [6]. Mitochondrial transfer, a newly discovered dynamic mitochondrial behavior, can respond to metabolic, oxidative, or genotoxic stress by maintaining metabolic homeostasis and intercellular communication in the tissue microenvironment [7]. Abnormal mitochondrial transfer has been linked to various human diseases, including cancer and cardiovascular diseases [8]. Mitochondrial transfer depends on the communication between donor and recipient cells, and tunnel nanotubes (TNTs) are widely recognized as the main intercellular platform for unidirectional and bidirectional mitochondrial exchange [9]. However, the role of mitochondrial transfer in HCC remains unknown.

High mobility group protein 1 (HMGB1) is a nucleoprotein involved in maintaining nucleosome integrity and promoting gene transcription in the nucleus. It can be actively secreted or passively released to play multiple roles as a damage-associated molecular pattern (DAMP) molecule in solid tumors of HCC, where hypoxia is particularly prominent, as well as in diseases like infections, endotoxemia, and ischemia-reperfusion events [10, 11]. Numerous studies have demonstrated that HMGB1 can regulate various aspects, including mitochondrial function and autophagy [12–14]. Previously, we discovered that HMGB1 can migrate to the cytoplasm and be released into the extracellular space under hypoxic conditions, where it can interact with the mitochondria to regulate the progression of HCC [15, 16]. However, the relationship between HMGB1 and mitochondrial transfer, the newly discovered mitochondrial kinetic behavior, and the associated mechanisms are currently unknown, which piqued our research interest.

In this study, we investigated whether mitochondrial transfer occurs between HCC cells, the involvement of TNTs, and the effects of mitochondrial transfer on HCC cells. Moreover, we explored the effects of hypoxia and HMGB1 on mitochondrial transfer and the specific underlying molecular mechanisms.

## Methods

### Clinical samples of human HCC

A total of 99 HCC patient samples were collected from Tongji Hospital, affiliated with Tongji Medical College of Tongji Medical College, and confirmed as HCC by the Department of Pathology. Informed consents from all patients participating in this study was obtained. Frozen tissues were used for immunoblotting analysis, while paraffin tissues were used for immunohistochemistry and immunofluorescence staining. This study was conducted according to the declaration of Helsinki of 1975 and approved by the Ethics Committee of Tongji Hospital.

### Bioinformatics analysis

Bioinformatics analysis was conducted on 420 samples (50 normal and 370 HCC samples) obtained from TCGA (https://portal.gdc.cancer.gov/repository) and 434 samples (202 normal and 232 HCC samples) obtained from ICGC (https://dcc.icgc.org/projects/LIRI-JP). The *t*-test was used to compare gene expression differences between HCC and adjacent normal tissues. Similar to Liang et al. [17–19]., we determined the relationship between gene expression and survival in HCC patients using survival R packets and KM analysis. Correlation analysis was used to determine the association between the genes.

### Cell culture

The cells were cultured in Dulbecco’s modified eagle medium (DMEM) containing 10% fetal bovine serum (FBS) at 37 °C in a humidified incubator containing 5% CO_2._ When cultured under hypoxic conditions, the cells were transferred to a chamber containing 1% O_2._ Human HCC cell lines PLC, MHCC-97H, and Hep3B were obtained from the Institute of Liver and Digestive Diseases, Tongji Hospital, Tongji Medical College. Cell lines were authenticated by STR analysis and tested for negative mycoplasma contamination.

### Lentivirus

The target gene overexpression lentivirus vector (pLVX-IRES neo), target gene knockdown lentivirus vector (pLKO.1 neo), luciferase lentivirus (Lenti-luciferase-P2A puro), red fluorescent virus (pLVX-mCherry puro), and green fluorescent lentivirus (pLVX-EGFP puro) required for the experiment were purchased from Wuhan Puyun Biotechnology Co. Ltd. Mito-PA-GFP puro, a green fluorescent protein lentivirus targeting mitochondrial matrix, was acquired from Obio (Shanghai) Technology Co., Ltd. The sh-sequence of the target gene knockdown lentivirus is included in the supplementary materials.

After 72 h of transfection with the corresponding lentivirus, G418 or puromycin was used to screen cells for about 14 days; the transfection efficiency was verified using WB assay analysis. When Lenti-luciferase-P2A puro was used, its transfection status was determined using live imaging equipment, while in the case of lentivirus with fluorescent elements, the transfection status was observed using a fluorescence microscope.

### Dyes and labeling of mitochondria

MitoTracker-Red (Cell Signaling Technology, 9082) was used for fluorescence staining of mitochondria, according to the manufacturer’s instructions [7, 20, 21]. Mito-PA-GFP lentivirus (green fluorescent protein targeting mitochondrial matrix) was used for fluorescent labeling of mitochondria in HCC cell culture [22].

### Transfection of siRNA

The siRNA required for the experiment was designed and synthesized by RiboBio (China), and the siRNA sequences are included in the supplementary materials. In a single well of the six-well plate, cells were seeded at a density of ~30%–40%, and 500 μL opti-MEM (Gibco), 5 μL siRNA, and 5 μL lipo3000 were added and incubated at room temperature for 20 min. Then, 1.5 mL of new serum-free medium was added to each well. After 8 h, DMEM supplemented with 10% FBS was added, and cell proteins were collected 72 h after transfection.

### Cell migration and invasion assay

An 8 μm Corning chamber adapted to 24 well plates was used to analyze the cell’s cross-pore migration and invasion ability. A serum-free cell suspension (200 μL) was seeded into the chamber, and a 600 μl culture medium containing 10% FBS was injected into the bottom chamber. After 24 or 48 h of incubation, the cells were fixed with 4% paraformaldehyde, stained with 0.1% crystal violet, and photographed under a microscope to analyze their migration ability. To assess the invasiveness of cells, based on these methods, each compartment was pre-coated with matrix gel (Corning Matrix Matrix) and serum-free DMEM in a ratio of 1:7 and incubated at 37 °C for 30 min to prepare a 50 μL mixture.

### Flow sorting and flow analysis

After cell counting, the cells were co-cultured under various conditions. The cells were digested and a cell suspension was prepared with a density of 2–5 million cells following co-culture. For flow sorting, the samples were sorted using BD FacsAriaIII. The required cells were collected in sterile flow cytometry tubes containing 10% FBS in DMEM and counted before the next experiment. Flow cytometry analysis was conducted using Beckman Coulter CytoFlex, and the data were processed using FlowJo software.

### Cell Counting Kit-8 (CCK-8) assay

Cell suspensions (1 × 10^3^ cells/well) were seeded in 96 well plates and incubated under normoxic and hypoxic conditions for 24 h. The absorbance was measured according to the CCK8 assay protocol (QSJ-001, Wuhan Promoter Biological Co., Ltd.).

### Immunofluorescence

Paraffin-embedded tissue samples were dewaxed and hydrated for antigen repair. Cell samples were fixed with 4% paraformaldehyde for 20 min. Paraffin tissue samples or cells were then treated with 0.3% Triton X 100 for 10 min and blocked with goat serum for 15 min. The samples were diluted with primary antibody and incubated at 4 °C overnight. Under dark conditions, the cells were stained with Actin-Green for 20 min, incubated with secondary fluorescent antibodies for 2 h, and then stained with DAPI for 30 min. The samples were observed and photographed using a fluorescence microscope.

### Immunohistochemistry

The experimental procedure has been described previously [23]. Three pathology teachers read the films using a double-blind method. IHC staining scores ≤5 indicated low expression, while 6–12 indicated high expression.

### Co-immunoprecipitation (CoIP)

Proteins were extracted from the treated cells using 1% NP40 and blocked with 50% protein A/G agarose. The primary antibody was added according to the manufacturer’s instructions and rotated using the magnetic bead overnight at 4 °C. The mixture was incubated with 50% protein A/G agarose and washed with the lysis solution. Agarose was resuspended in a protein loading buffer, boiled at 100 °C for 10 min, and then WB analysis was performed.

### Chromatin immunoprecipitation (ChIP)

The cells were cross-linked with formaldehyde and treated with ultrasound. The segmented chromatin was incubated with the target antibody or anti-IgG antibody to capture and separate the DNA-protein complex. After de-crosslinking and DNA purification, all DNA samples were subjected to qRT-PCR analysis to quantify the corresponding binding sites on the promoter. The primer sequences are listed in the Supplementary Table. The experiment was repeated at least three times.

### Luciferase reporter assay

The firefly luciferase reporter plasmid was co-transfected with the Renilla luciferase reporter plasmid for detection. The ratio of firefly and rinilla luciferase activity was measured after 48 h of transfection using a double Luciferase ® Reporter gene measurement system (Vazyme Biotech Co., Ltd).

### Extraction of membrane and cytoplasmic proteins

Membrane and cytoplasmic proteins were extracted using the KeyGEN BioTECH Membrane Protein and Cytoplasmic Protein Kit, according to the manufacturer’s instructions.

### Western blot (WB) assay

After extraction of proteins from tissue samples and processed cell samples, the protein concentration was measured using the BCA kit (Wuhan Servicebio Technology Co. Ltd). Loading buffer (Wuhan Servicebio Technology Co. Ltd) was added, boiled at 100 °C for 10 min, and stored at –80 °C. The proteins were separated using 8%–12% SDS gel electrophoresis and transferred to a 0.22 μm polyvinylidene fluoride (PVDF) membrane. The strip was blocked with 5% skimmed milk or 5% BSA at room temperature for 2 h, diluted at 4 °C for 12 h per the instructions of the primary antibody, incubated with the second antibody at room temperature for 2 h, and then exposed.

### Quantitative real-time polymerase chain reaction (qRT-PCR)

Total RNA was extracted from cells using trizol reagent (Invitrogen) and quantified to 1.0 μg/μL using Thermo Scientific NanoDrop 2000. According to the manufacturer’s instructions, the PrimeScript reagent kit (Takara) was used to synthesize the first chain cDNA. Real-time PCR analysis of β-actin mRNA was conducted using SYBR Premix ExTaq (Vazyme Biotech Co., Ltd.) for target genes and internal references. The specific primers are shown in the supplementary materials.

### Animal model

Five-week-old male thymus-free BALB/c mice were purchased and raised under pathogen-free conditions. The mice were randomly assigned to different groups, with 6 mice in each group. All animal experiments were conducted according to the protocol approved by the Ethics Review Committee of Tongji Medical College of Tongji Medical College. We established a tail vein lung metastasis model to study the metastasis ability of HCC cells and injected mice with diluted PBS containing 1 × 10^6^ cells through the tail vein. After ~6 weeks, a bioluminescence imaging system was used to confirm the presence of metastasis in mice. After the animals were euthanized, the metastatic nodules in the lungs were detected using hematoxylin and eosin staining.

### Data analysis

All experiments were repeated more than three times. *P* < 0.05 is considered statistically significant. *P < 0.05; **P < 0.01; ***P < 0.001. The Kaplan–Meier method (logarithmic rank test) was used to investigate the overall survival rate. The Pearson correlation analysis was used to analyze the correlation between the expression of the two molecules. The *P*-values of quantitative data were analyzed using Student’s *t*-test or ANOVA, while the *P*-values of categorical data were analyzed using the *χ*^*2*^ test or Fisher’s exact test.

## Results

### Hypoxia promotes mitochondrial transfer between HCC cells

**Fig. 1:**
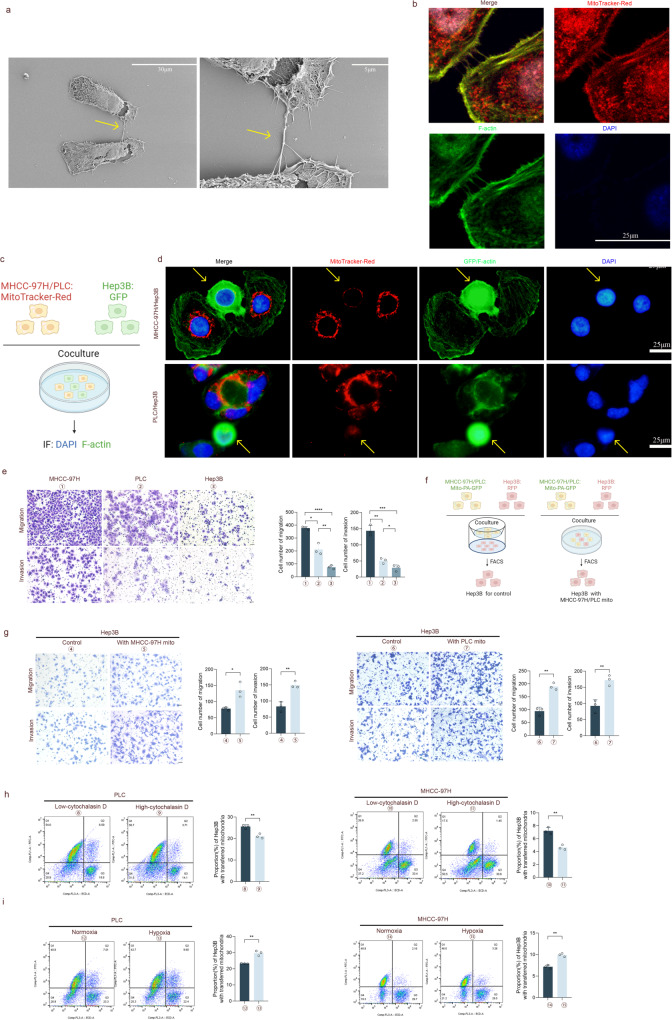
Hypoxia promotes mitochondria transfer between HCC cells. **a** Scanning electron microscopy image showing the presence of nanotunnels between the two co-cultured HCC cells. Scale bars: 30 and 5 μm. **b** Immunofluorescence showing the nano tunnels (green) and mitochondria (red) between the two co-cultured HCC cells. Scale bars: 25 μm. **c** Schematic diagram of MHCC-97H and PLC cells labeled with MitoTracker-Red and co-cultured with GFP-labeled Hep3B cells for immunofluorescence staining. **d** Immunofluorescence staining: Red fluorescence signal (MitoTracker-Red), green fluorescence signal (GFP and F-actin), and blue fluorescence signal (DAPI). Scale bars: 25 μm. **e** The migration and invasion abilities of MHCC-97H, PLC, and Hep3B cells were compared using a Transwell chamber. The total number of cells in the cell suspension of all three cells was 60000, and the treatment time was 24 h. Scale bars: 50 μm. *n* = 3, *P < 0.05, **P < 0.01, ***P < 0.001, ****P < 0.0001. **f**, **g** The schematic diagram of MHCC-97H and PLC cells labeled with Mito-PA-GFP, co-cultured with Hep3B cells labeled with RFP, and subjected to sterile cell flow cytometry sorting, followed by metastasis and invasion experiments. Scale bars: 50 μm. *n* = 3, *P < 0.05, **P < 0.01. **h** MHCC-97H and PLC cells labeled with Mito-PA-GFP were co-cultured with Hep3B cells labeled with RFP at different concentrations (0.2 μg/mL, 0.8 μg/mL) of cytochalasin D for 24 h, and then cell flow analysis was conducted to determine the proportion of Hep3B cells containing transferred mitochondria. *n* = 3, **P < 0.01. **i** MHCC-97H and PLC cells labeled with Mito-PA-GFP were co-cultured with Hep3B cells labeled with RFP under normoxic and hypoxic conditions for 24 h, and flow cytometry was performed to determine the proportion of Hep3B cells containing transferred mitochondria. *n* = 3, **P < 0.01.

The interaction between co-cultured HCC cells was examined using a field emission scanning electron microscope, and it was discovered that a physical connection between HCC cells could be made through a nanoscale tubular structure (Fig. 1a). MitoTracker-Red was used to label the mitochondria while phalloidin green was used to stain F-actin in the nanotubes. Co-localization of MitoTracker-labeled mitochondria in the nanotubes was observed using immunofluorescence (IF) (Fig. 1b). The mitochondria in MHCC-97H cells were labeled with MitoTracker-Red dye, thoroughly washed to remove the unbound dye, and then co-cultured with a green fluorescent protein (GFP)-labeled Hep3B cells for 24 h. DAPI nuclear staining and phalloidin green F-actin staining were performed (Fig. 1c). The individual fluorescence images of GFP and the results of individual phalloidin green F-actin staining were shown in Fig. S1a. The yellow arrow in Fig. 1d shows the mitochondria labeled with MitoTracker-Red dye from MHCC-97H cells in GFP-labeled Hep3B cells. The same phenomenon was observed in the co-culture of PLC and Hep3B cells (Fig. 1c, d), indicating that mitochondria can transfer between HCC cells through TNTs.

To analyze the role of mitochondrial transfer between HCC cells, we compared the invasion and metastasis abilities of three commonly used HCC cell lines, MHCC-97H, PLC, and Hep3B, using Transwell migration and invasion experiments (Fig. 1e). Hep3B cells were identified to be relatively lower invasive HCC cells. The mitochondria in MHCC-97H and PLC cells were labeled with Mito-PA-GFP (Fig. S1b). Then, Mito-PA-GFP labeled MHCC-97H and PLC cells were co-cultured with red fluorescence protein (RFP) labeled Hep3B cells for 24 h, respectively (Fig. 1f), and fluorescence-activated cell sorting (FACS) was used to identify Hep3B cells containing MHCC-97H mitochondria or PLC mitochondria as the experimental group [24]. After sorting, Hep3B cells were subjected to transwell migration and invasion experiments (Fig. 1g). The results showed that Hep3B cells containing MHCC-97H or PLC mitochondria had improved migration and invasion abilities. However, there was no statistically significant difference in the migration and invasion abilities of MHCC-97H cells and PLC cells containing Hep3B mitochondria (Fig. S1c, d). This indicates that the transfer of mitochondria from highly invasive HCC cells to less invasive HCC cells may enhance their metastatic and invasive abilities.

The rate of mitochondrial transfer was quantified using a co-culture system of MHCC-97H and PLC cells labeled with Mito-PA-GFP and Hep3B cells labeled with RFP to analyze the proportion of FITC^+^ ECD^+^ Hep3B cells to ECD^+^ Hep3B cells. Cytochalasin D can inhibit the synthesis of TNT [25–28]. We added different concentrations of cytochalasin D into co-culture (Fig. 1h). At higher concentrations of cytochalasin D, the mitochondrial transfer rate decreased. HCC, as a solid tumor, has always exhibited hypoxia in its microenvironment [29]. Therefore, we treated the co-cultured cells under normal and hypoxic conditions for 24 h and discovered that the rate of mitochondrial transfer increased under hypoxic conditions (Figs. 1i and S1e). In addition, we observed TNTs between HCC cells under normoxic and hypoxic conditions using confocal. As shown in Fig. S1f, the number of TNT between HCC cells increased under hypoxic conditions. This indicates that hypoxia can promote the mitochondrial transfer between HCC cells.

### HMGB1 promotes mitochondrial transfer and the development of HCC cells

**Fig. 2:**
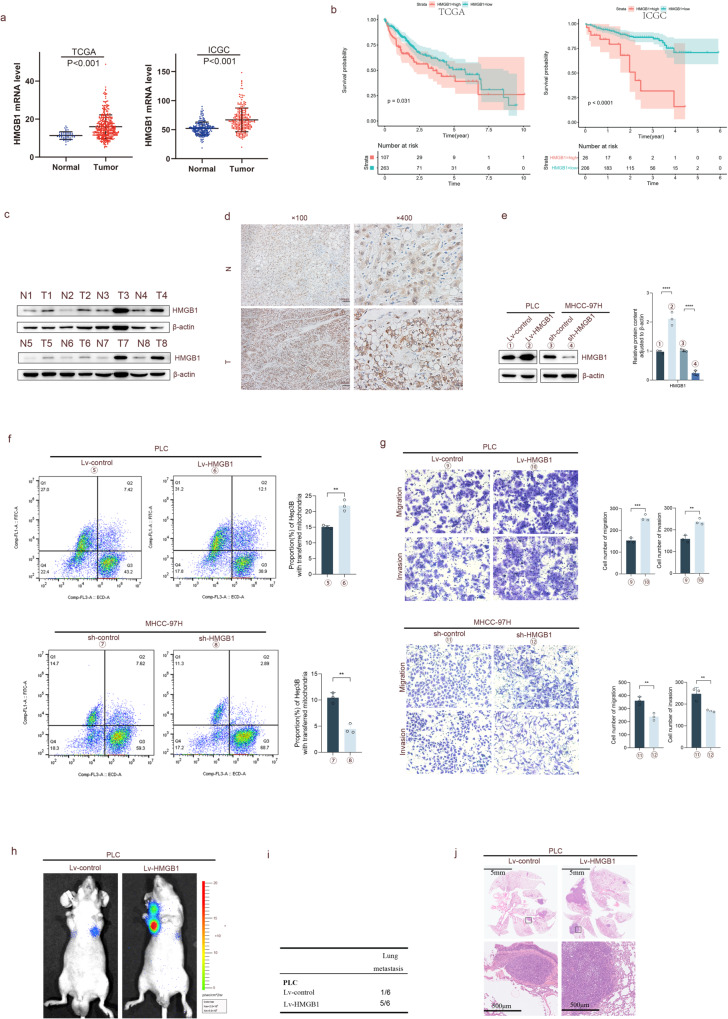
HMGB1 promotes mitochondrial transfer and the development of HCC cells. **a**, **b** Bioinformatics analysis of HMGB1 mRNA expression and survival curve of TCGA and ICGC databases. **c** WB analysis of HMGB1 protein expression in eight pairs of HCC tissues and adjacent non-tumor tissues. **d** Immunohistochemical analysis of the localization and expression of HMGB1 in HCC and adjacent non-tumor tissues. Scale bars: 100 and 25 μm. **e** WB analysis of HMGB1 protein levels after transfection of PLC and MHCC-97H cells with high and low expression lentiviruses, respectively. *n* = 3, ****P < 0.0001. **f** MHCC-97H and PLC cells transfected with Mito-PA-GFP and HMGB1 lentivirus were co-cultured with RFP-labeled Hep3B cells for 24 h, and flow cytometry was performed to determine the proportion of Hep3B cells containing transferred mitochondria. *n* = 3, **P < 0.01. **g** The ability of HMGB1 lentivirus transfection into MHCC-97H and PLC cells to induce metastasis and invasion was analyzed using a transwell assay. *n* = 3, **P < 0.01, ***P < 0.001. **h**–**j** Lung metastasis experiments were conducted in a mouse model by injection into the tail vein, and representative luciferase signal images (**h**), lung metastasis statistical tables (**i**), and representative H&E staining of lung tissue (**j**) were obtained. Scale bars: 5 mm and 500 μm.

The following experiments were conducted to confirm the role of HMGB1 in mitochondrial transfer, as well as the migration and invasion of HCC. According to data from TCGA and ICGC (Fig. 2a, b), the mRNA expression level of HMGB1 was significantly higher in HCC tissues than in adjacent healthy tissues. HCC patients with increased expression of HMGB1 had a shorter overall survival period. WB analysis revealed that the protein concentration of HMGB1 was higher in HCC tissues than in adjacent healthy tissues (Fig. 2c). Immunohistochemistry staining revealed that HMGB1 was more localized in the nucleus in the adjacent healthy tissues, and the cytoplasm was stained deeper in the HCC tissues (Fig. 2d).

To elucidate the role of HMGB1 in mitochondrial transfer and the invasion and metastasis of HCC cells, we constructed cell lines with overexpression and knockdown of HMGB1 using lentivirus vector transfection and antibiotic screening. The related protein expression is illustrated in Fig. 2e. First, we determined the effects of changes in HMGB1 expression on the mitochondrial transfer rate using the co-culture model in Fig. 1h for 24 h. The results revealed that HMGB1 overexpression could promote mitochondrial transfer, while HMGB1 knockdown could reduce the transfer (Fig. 2f). HMGB1 overexpression enhanced the migration and invasion ability of HCC cells, while the knockdown decreased their ability (Fig. 2g). Similar results were observed in the tail vein lung metastasis experiment in nude mice (Fig. 2h–j), showing that the HMGB1 overexpression group had stronger biological signals in the lung metastatic lesions during in vivo imaging, a relatively higher incidence of lung metastasis, and a larger area of lung metastatic lesions.

### RHOT1 promotes mitochondrial transfer and the metastasis and invasion of HCC cells

**Fig. 3:**
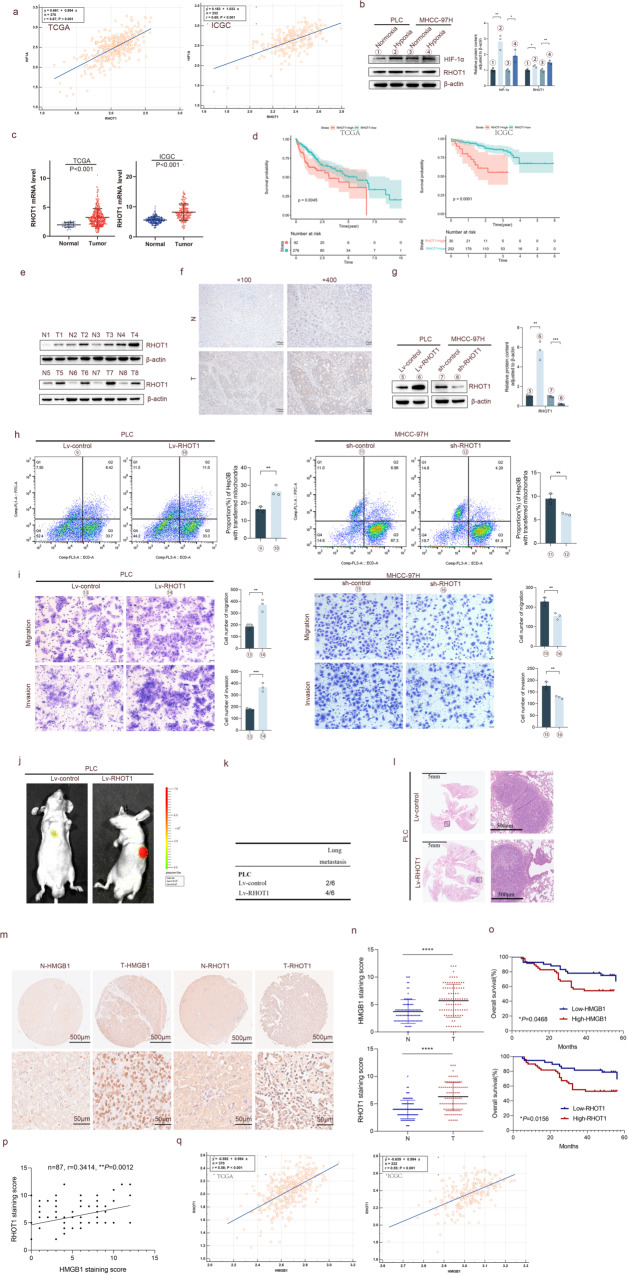
RHOT1 promotes mitochondrial transfer and metastasis and invasion of HCC cells. **a** Bioinformatics analysis of the correlation between RHOT1 and HIF1α on TCGA and ICGC databases. **b** WB analysis of the changes in RHOT1 in PLC and MHCC-97H cells after 24 h of normoxic and hypoxic conditions. *n* = 3, *P < 0.05, **P < 0.01. **c**, **d** Bioinformatics analysis of RHOT1 mRNA expression and survival curve of TCGA and ICGC databases. **e** WB analysis of RHOT1 protein expression in eight pairs of HCC and adjacent non-tumor tissues. **f** Immunohistochemical analysis of the localization and expression of RHOT1 in HCC and adjacent non-tumor tissues. Scale bars: 100 and 25 μm. **g** WB analysis of RHOT1 protein levels after transfection of PLC and MHCC-97H cells with lentiviruses. *n* = 3, *P < 0.05, **P < 0.01, ***P < 0.001. **h** MHCC-97H and PLC cells transfected with Mito-PA-GFP and RHOT1 lentiviruses were co-cultured with RFP-labeled Hep3B cells for 24 h, and flow cytometry was performed to determine the proportion of Hep3B cells containing transferred mitochondria. *n* = 3, **P < 0.01. **i** Cell migration and invasion assay after RHOT1 lentivirus transfection into MHCC-97H and PLC cells. Scale bars: 50 μm. *n* = 3, **P < 0.01, ***P < 0.001. **j**–**l** Lung metastasis experiments were conducted on a mouse model through injection into the tail vein, and representative luciferase signal images (**j**), lung metastasis statistical tables (**k**), and representative hematoxylin and eosin (H&E) staining of lung tissue (**l**) were obtained. Scale bars: 5 mm and 500 μm. **m** Representative images of immunohistochemistry staining of HMGB1 and RHOT1 tissue microarrays. Scale bars: 500 and 50 μm. **n** Immunohistochemistry scores of HMGB1 and RHOT1 in tissue microarrays. ****P < 0.0001. **o** Kaplan–Merier analysis of overall survival of HMGB1 and RHOT1 in tissue microarrays. **p** correlation analysis of HMGB1 and RHOT1 expression in tissue microarrays. **q** Correlation analysis of HMGB1 and RHOT1 in TCGA and ICGC databases.

The spatial location and movement of mitochondria are mainly regulated by RHOT1 (also called Miro1), which belongs to the Miro protein of the small GTPase subfamily and is located in the outer membrane of the outer mitochondrial membrane [30]. RHOT1 expression is upregulated in pancreatic cancer, cholangiocarcinoma, esophageal cancer, glioblastoma, acute myeloid leukemia, thymoma, and other cancers [31]. As HIF1A is a crucial molecule closely related to hypoxia [32], we first analyzed the relationship between RHOT1 and HIF1A using bioinformatics to investigate the expression of RHOT1 under hypoxia in advance. The data of TCGA and ICGC (Fig. 3a) revealed that the correlation coefficients between the two were 0.67 and 0.60, respectively, with *P* < 0.001. Therefore, WB assay was conducted to analyze the protein expression of RHOT1 in PLC and MHCC-97H cells before and after hypoxia; the expression of RHOT1 increased after hypoxia (Fig. 3b). According to the data from TCGA and ICGC (Fig. 3c, d), the mRNA expression level of RHOT1 in HCC tissues was significantly higher than that in the adjacent healthy tissues. However, HCC patients with increased expression of RHOT1 had a shorter overall survival period. Subsequently, WB assay of the clinical samples revealed that the protein level of RHOT1 was higher in HCC than in the adjacent healthy tissues (Fig. 3e). Immunohistochemistry staining revealed that RHOT1 was more localized in the cytoplasm of cells, and HCC staining was deeper compared to the matched adjacent non-tumor tissues (Fig. 3f).

Similarly, we constructed RHOT1 overexpression and knockdown cell lines using lentivirus vector transfection and antibiotic screening. The associated protein expression is displayed in Fig. 3g. RHOT1 overexpression could promote mitochondrial transfer, while RHOT1 knockdown could reduce the transfer (Fig. 3h). Moreover, RHOT1 overexpression enhanced the migration and invasion abilities of HCC cells, whereas RHOT1 knockdown decreased these abilities (Fig. 3i). Similar results were observed in the tail vein lung metastasis experiment in nude mice (Fig. 3j–l) that the RHOT1 overexpression group exhibited stronger biological signals for the lung metastatic lesions during in vivo imaging, a relatively higher incidence of lung metastasis, and a larger area of lung metastatic lesions.

To analyze the relationship between HMGB1 and RHOT1 in clinical samples further, we used tissue microarrays for immunohistochemistry staining, including 87 paired HCC tissue samples. Similar to the results of bioinformatics, the expression of HMGB1 and RHOT1 was higher in HCC tissues than in the adjacent healthy tissues (Fig. 3m–o). HCC patients with increased expression of HMGB1 and RHOT1 had a shorter overall survival period. Moreover, we conducted a correlation analysis on the expression of HMGB1 and RHOT1 and discovered a positive correlation (*n* = 87, *r* = 0.3414, *P* = 0.0012) between the two in HCC patients in the tissue microarray (Fig. 3p). Similarly, in the data from TCGA (*n* = 370, *r* = 0.58, *P* < 0.001) and ICGC (*n* = 232, *r* = 0.55, *P* < 0.001), the two were positively correlated (Fig. 3q).

### HMGB1 enhances RHOT1 expression through NFYA and NFYC subunits in the NF-Y complex under hypoxia

**Fig. 4:**
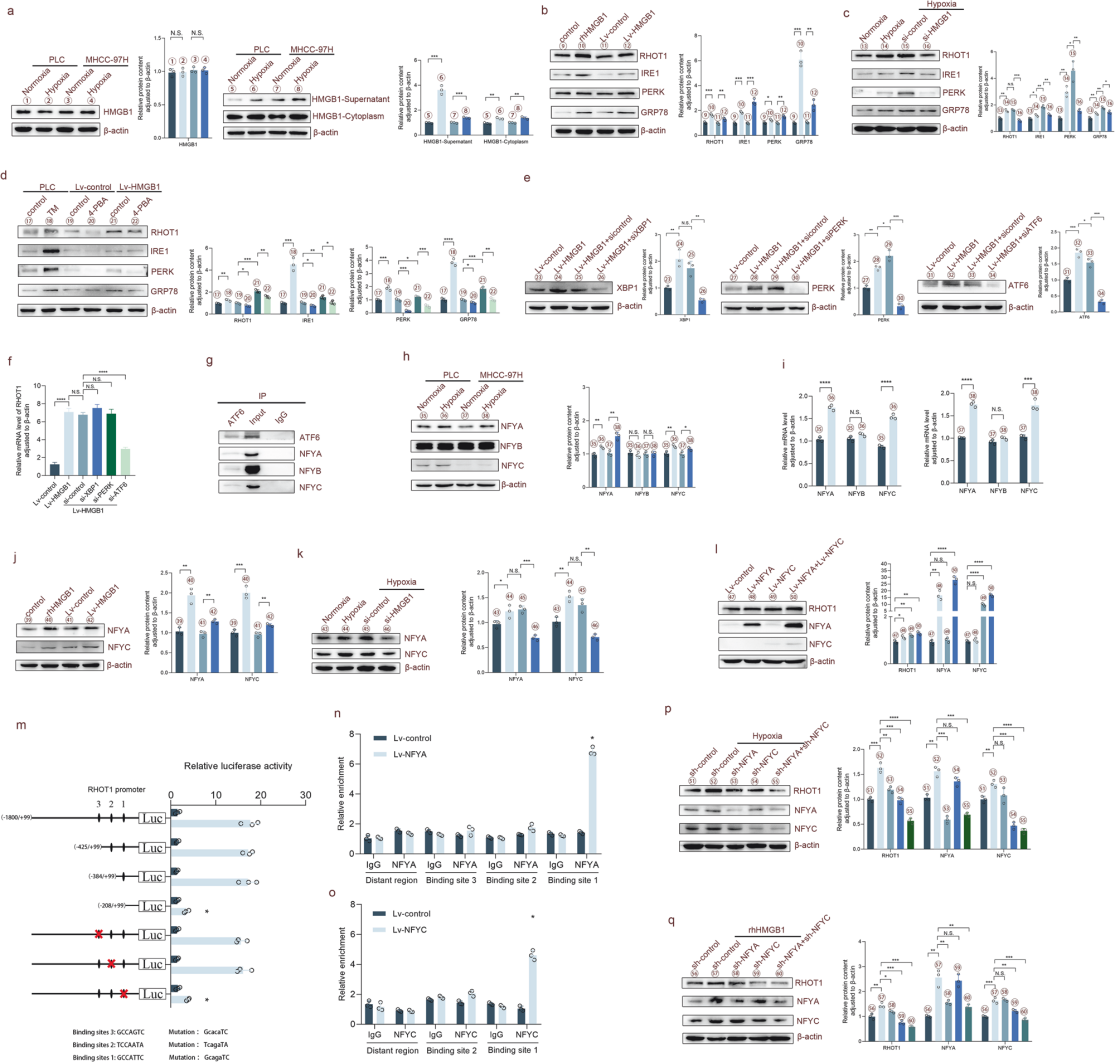
HMGB1 enhances RHOT1 expression through NFYA and NFYC subunits in the NF-Y complex under hypoxia. **a** WB analysis of HMGB1 protein content in the cell lysate, cytoplasm, and supernatant of PLC and MHCC-97H cells after 24 h of normoxia and hypoxia. *n* = 3, **P < 0.01, ***P < 0.001. N.S: no significance. **b** WB analysis showing the protein content of RHOT1, IRE1, PERK, and GRP78 in PLC cells after stimulation with recombinant human HMGB1 protein (1 μg/mL, 24 h) and overexpression of HMGB1 after lentivirus transfection. *n* = 3, *P < 0.05, **P < 0.01, ***P < 0.001. **c** WB analysis of the protein content of IRE1, PERK, and GRP78 in PLC cells after 24 h of normoxic and hypoxic conditions, as well as after treatment with si-HMGB1 under hypoxic conditions. *n* = 3, *P < 0.05, **P < 0.01. **d** WB analysis of the protein content of RHOT1, IRE1, PERK, and GRP78 in PLC cells treated with 4-PBA (5 mM, 24 h), TM (0.5 μg/mL, 24 h), and HMGB1 overexpression lentivirus transfection. *n* = 3, *P < 0.05, **P < 0.01, ***P < 0.001, ****P < 0.0001. **e**, **f** WB analysis of PLC cells overexpressing HMGB1 and transfected with siRNA. Protein content of XBP1, PERK, and ATF6 and mRNA level analysis of RHOT1 under different treatment conditions. *n* = 3, *P < 0.05, **P < 0.01, ***P < 0.001. N.S.; no significance. **g** The CoIP results revealed that ATF6 can interact with NFYA, NFYB, and NFYC. **h**, **i** Protein content and mRNA levels of NFYA, NFYB, and NFYC under hypoxic conditions. *n* = 3, *P < 0.05, **P < 0.01, ****P < 0.0001. N.S.; no significance. **j** WB analysis showing changes in the protein content of NFYA and NFYC in PLC cells after stimulation with recombinant human HMGB1 protein and HMGB1 overexpression after lentivirus transfection. *n* = 3, **P < 0.01, ***P < 0.001. **k** Protein content of RHOT1, NFYA, and NFYC in PLC cells after 24 h of normoxic and hypoxic conditions, as well as treatment with si-HMGB1 under hypoxic conditions. *n* = 3, *P < 0.05, **P < 0.01, ***P < 0.001. N.S. no significance. **l** WB analysis of the protein content of RHOT1, NFYA, and NFYC overexpressed in PLC cells after lentivirus transfection in NFYA and NFYC. *n* = 3, *P < 0.05, **P < 0.01, ****P < 0.0001. N.S. no significance. **m** PLC cells were transfected with NFYA and NFYC overexpressing lentivirus, then with truncated and mutant plasmids designed according to the RHOT1 promoter region, and luciferase activity was measured. **n**–**o** The ChIP experiment verifying the direct binding sites of NFYA and NFYC to the RHOT1 promoter in PLC cells. **p** WB analysis showing changes in protein content of RHOT1, NFYA, and NFYC in PLC cells after knocking down NFYA and NFYC under hypoxic conditions. *n* = 3, **P < 0.01, ***P < 0.001, ****P < 0.0001. N.S. no significance. **q** WB analysis showing changes in protein concentrations of RHOT1, NFYA, and NFYC in PLC cells after knocking down NFYA and NFYC under stimulation of recombinant human HMGB1 protein. *n* = 3, *P < 0.05, **P < 0.01, ***P < 0.001. N.S. no significance.

Our previous study revealed that hypoxia could promote the release of HMGB1 from HCC cells, which interacts with mitochondria to promote tumor progression [15]. Consistent with our previous results, hypoxia induced HMGB1 translocation to the cytoplasm and extracellular space (Figs. 4a and S2a). The biological behavior of mitochondria is closely related to the regulation of endoplasmic reticulum, and endoplasmic reticulum stress plays a key role in the communication between endoplasmic reticulum and mitochondria [33]. Our previous research revealed that HMGB1 can induce endoplasmic reticulum stress [16]. Therefore, we investigated whether HMGB1 could regulate RHOT1 expression through endoplasmic reticulum stress. We identified the changes in RHOT1 and endoplasmic reticulum stress signaling pathway through WB analysis after the PLC cells were stimulated by recombinant human HMGB1 protein and high expression of HMGB1. Stimulation with recombinant human HMGB1 protein and HMGB1 overexpression increased RHOT1 protein expression and activated the endoplasmic reticulum stress signaling pathway. PLC cells were subsequently placed under hypoxic conditions and subjected to various combinations of si-HMGB1 treatment (Fig. 4c). The results revealed that the expression of RHOT1, IRE1, PERK, and GRP78 increased under hypoxia, and their overexpression was inhibited after si-HMGB1 treatment.

To confirm the specific effects of endoplasmic reticulum stress on RHOT1, PLC cells and HMGB1 overexpressed cells were treated with the activator tunicamycin (TM) and the inhibitor 4-PBA, respectively (Fig. 4d). Treatment with TM increased RHOT1 expression, whereas 4-PBA inhibited it. The perception and response to endoplasmic reticulum stress are coordinated by the unfolded protein response (UPR), which has three endoplasmic reticulum membrane-embedded sensors: double-stranded RNA-dependent protein kinase-like ER kinase (PERK), activated transcription factor 6 (ATF6), and inositol-requiring enzyme 1 (IRE1) [34]. To identify the specific regulatory mechanism of endoplasmic reticulum stress on RHOT1, we used siRNA to inhibit the key molecules X-box binding protein 1 (XBP1), PERK, and ATF6 of the three pathways. Figure 4e indicates that the specific inhibitory effects were evident. The changes in RHOT1 mRNA levels after treatment with the three molecules are depicted in Fig. 4f, indicating that after treatment with si-ATF6, the changes in RHOT1 expression were statistically significant. Several studies have shown that ATF6α or ATF6β can combine with the NF-Y complex to form heterologous protein complexes when the cell UPR is activated [35]. After analyzing various bioinformatics websites, including JASPAR (https://jaspar.genereg.net), it was discovered that the NF-Y complex might be the transcription factor of RHOT1. Therefore, co-immunoprecipitation (CoIP) experiments were conducted using ATF6, and the NF-Y transcription factor complex was identified. Subsequently, specific protein and mRNA changes were detected in the three components of NF-Y in PLC and MHCC-97H cells under hypoxic conditions, and the expression of NFYA and NFYC was increased (Fig. 4h). The expression of NFYA and NFYC increased in response to hypoxia and HMGB1(Fig. 4j, k). Therefore, we constructed HCC cells overexpressing NFYA and NFYC using lentiviruses (Fig. 4l), which could enhance the expression of RHOT1. According to the luciferase reporter assay (Fig. 4m) and chromatin immunoprecipitation (ChIP) experiment (Fig. 4n–o), NFYA and NFYC regulated the expression of RHOT1 by binding to the first binding site of the RHOT1 promoter. After inhibiting the expression of NFYA and NFYC under hypoxia and HMGB1 stimulation, RHOT1 expression could be inhibited (Fig. 4p, q). In conclusion, our results revealed that HMGB1 increased RHOT1 expression through the NFYA and NFYC subunits of the NF-Y complex under hypoxia.

### RAC1 binds to RHOT1 and aggregates to the cell membrane under the regulation of HMGB1 under hypoxia

**Fig. 5:**
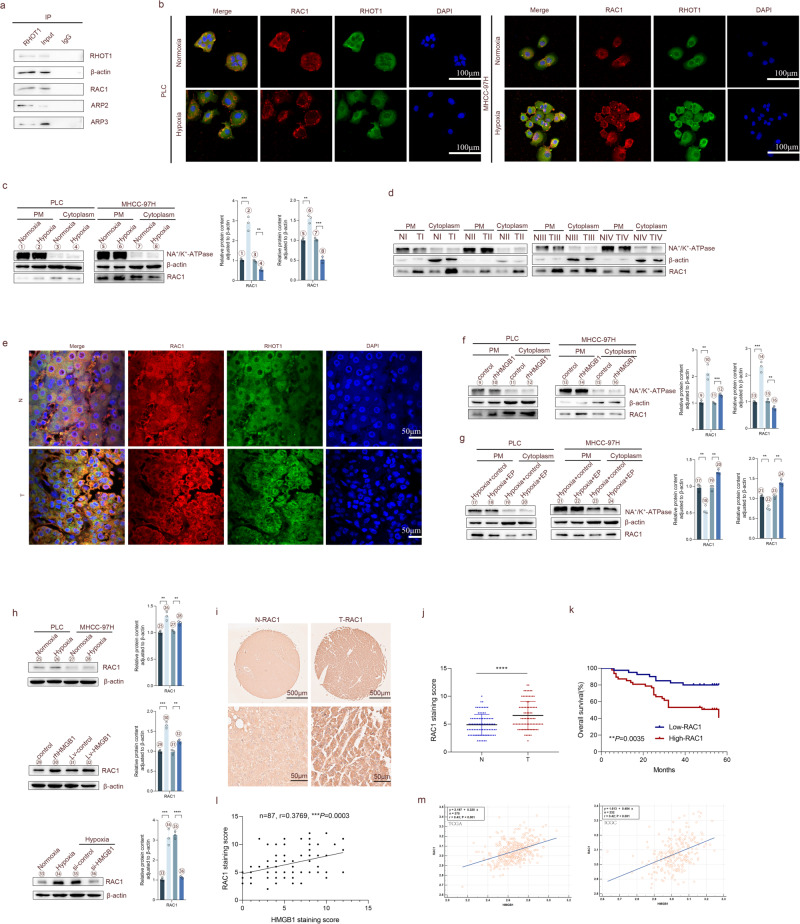
RAC1 binds to RHOT1 and aggregates to the cell membrane under HMGB1 regulation under hypoxia. **a** Co-IP results indicate that RHOT1 interacts with β-actin, RAC1, and ARP2/3. **b** Immunofluorescence staining to analyze the cellular localization of RHOT1 and RAC1 under normoxic and hypoxic conditions. Scale bars: 100 μm. **c** WB analysis of RAC1 protein content in the plasma membrane (PM) and cytoplasm of PLC and MHCC-97H cells under normoxic and hypoxic conditions. *n* = 3, **P < 0.01, ***P < 0.001. **d** WB analysis of RAC1 protein content in the plasma membrane and cytoplasm of HCC and adjacent non-tumor tissues. **e** Immunofluorescence staining was used to analyze the cellular localization relationship between RHOT1 and RAC1 in HCC and adjacent non-tumor tissues. Scale bars: 50 μm. **f** WB analysis of RAC1 protein content in the plasma membrane and cytoplasm of PLC and MHCC-97H cells stimulated with recombinant human HMGB1 protein. **P < 0.01, ***P < 0.001. **g** WB analysis of RAC1 protein content in the cell membrane and cytoplasm after inhibition of HMGB1 secretion by PLC and MHCC-97H cells using EP (10 mM, 24 h) under hypoxic conditions. *n* = 3, **P < 0.01. **h** RAC1 protein content in the whole lysate was determined under hypoxic conditions after stimulation with recombinant human HMGB1 protein and treatment with si-HMGB1 under hypoxic conditions for PLC and MHCC-97H. *n* = 3, **P < 0.01, ***P < 0.001, ****P < 0.0001. **i** Representative image of immunohistochemistry staining of RAC1 tissue microarray. Scale bars: 500 and 50 μm. **j** Immunohistochemistry score of RAC1 in tissue microarray. ****P < 0.0001. **k** Kaplan–Meier analysis of the correlation between RAC1 expression and overall survival in titanium microarrays. **l** Correlation analysis of HMGB1 and RAC1 in tissue microarrays. **m** Correlation analysis of HMGB1 and RAC1 in TCGA and ICGC databases.

The mechanism of TNT formation is significantly associated with the interactions between protein complexes [36]. To investigate the specific mechanism of TNT in mitochondrial transfer, we conducted CoIP experiments using RHOT1 and protein liquid chromatography-tandem mass spectroscopy. The results revealed that RAC1, ARP2/3, and β-actin could interact with RHOT1 (Fig. 5a). RAC1 belongs to the Ras superfamily and Rho family of small GTPases [37], mainly involved in the reorganization of the actin cytoskeleton, which can promote the formation of TNTs through the ARP2/3 complex [38]. The immunofluorescence experiments for RAC1 and RHOT1 were performed on HCC cells before and after hypoxia (Fig. 5b). A fluorescence co-localization was observed between the two. Simultaneously, RAC1 was relatively uniform in the cytoplasm of cells under normoxia; however, the fluorescence signal aggregated on the cell membrane under hypoxia. Protein extraction from the cell membrane of cancer cells before and after hypoxia revealed that the RAC1 content increased in the cell membrane after hypoxia (Fig. 5c). The WB and IF results indicated similar phenomena in HCC tissues (Fig. 5d, e). As shown in Fig. S2b, recombinant human HMGB1 protein increased the number of TNTs between HCC cells. Due to the phenomenon of HMGB1 translocation after hypoxia (Fig. 4a and S2a), it is of great interest whether the translocation of RAC1 is associated with HMGB1. Therefore, we stimulated HCC cells using recombinant human HMGB1 protein and treated them with HMGB1 translocation inhibitor (ethyl pyruvate, EP) under hypoxia [39]. The results revealed that HMGB1 could regulate the aggregation of RAC1 onto the cell membrane under hypoxic conditions (Figs. 5f, g and S2c). Moreover, HMGB1 could promote RAC1 expression under hypoxic conditions (Fig. 5h).

To further analyze the relationship between HMGB1 and RAC1 in clinical samples, immunohistochemistry staining of tissue microarrays was performed. Similar to the results of bioinformatics analysis, RAC1 expression was higher in HCC patients than in adjacent healthy tissues (Fig. 5i–k). HCC patients with high RAC1 expression had a shorter overall survival period. The correlation analysis of HMGB1 and RAC1 expression revealed a positive correlation (*n* = 87, *r* = 0.3769, *P* = 0.0003) between the two in HCC patients (Fig. 5l). Similarly, in the data of TCGA (*n* = 370, *r* = 0.43, *P* < 0.001) and ICGC (*n* = 232, *r* = 0.42, *P* < 0.001), the two were exhibited a positive correlation (Fig. 5m).

### HMGB1 promotes mitochondrial transfer through RHOT1 and RAC1 in HCC cells

**Fig. 6:**
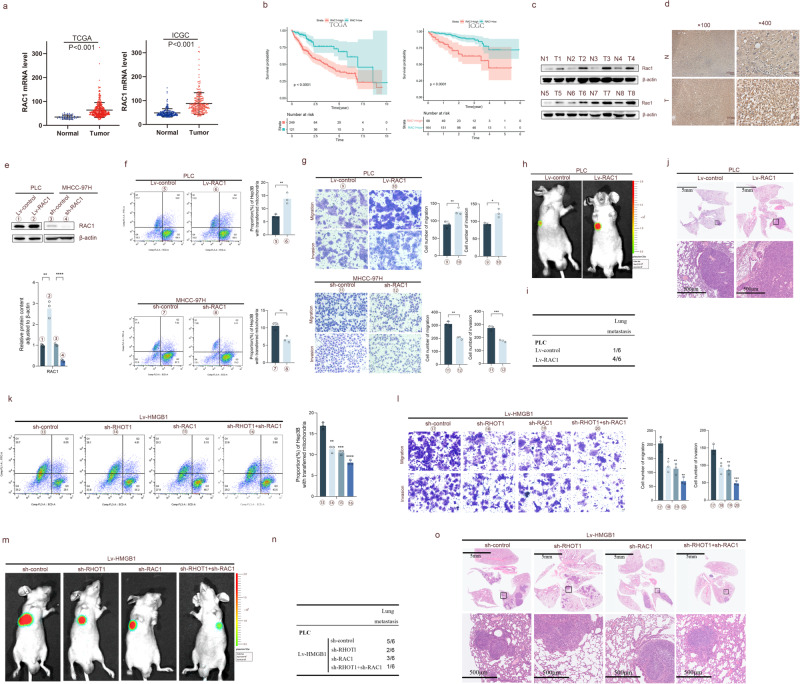
HMGB1 promotes mitochondrial transfer through RHOT1 and RAC1 in HCC cells. **a**, **b** Bioinformatics analysis of RAC1 mRNA expression and survival curve of TCGA and ICGC databases. **c** WB analysis of RAC1 protein expression in eight pairs of HCC and adjacent non-tumor tissues. **d** Immunohistochemical analysis of RAC1 expression and localization in HCC and adjacent non-tumor tissues. Scale bars: 100 and 25 μm. **e** WB analysis of RAC1 protein levels after lentivirus transfection into PLC and MHCC-97H cells. *n* = 3, **P < 0.01, ****P < 0.0001. **f** MHCC-97H and PLC cells transfected with Mito-PA-GFP and RAC1 lentivirus were co-cultured with RFP-labeled Hep3B cells for 24 h, and flow cytometry was performed to determine the proportion of Hep3B cells containing transferred mitochondria. *n* = 3, **P < 0.01. **g** The ability of RAC1 lentivirus transfection into MHCC-97H and PLC cells for metastasis and invasion was analyzed using transwell assay. Scale bars: 50 μm. *n* = 3, *P < 0.05, **P < 0.01, ***P < 0.001. **h**–**j** Lung metastasis experiments were conducted in a mouse model through injection into the tail vein, and representative luciferase signal images (**h**), lung metastasis statistical tables (**i**), and H&E staining images of lung tissue (**j**) were obtained. Scale bars: 5 mm and 500 μm. **k**–**o** Changes in mitochondrial transfer (**k**), transwell cell migration and invasion ability (**l**), and tail vein mouse lung metastasis model (**m**–**o**) after knocking down RHOT1 and RAC1 in PLC cells stably transfected with HMGB1. Scale bars: 5 mm and 500 μm. All *P*-values were compared with those of the Lv-HMGB1-sh-control group. *n* = 3, *P < 0.05, **P < 0.01, ***P < 0.001, ****P < 0.0001.

The following experiments were conducted to determine the role of RAC1 in mitochondrial transfer and the migration and invasion of HCC. TCGA and ICGC data (Fig. 6a, b) indicated that the mRNA expression level of RAC1 was significantly higher in HCC tissues than in adjacent normal tissues. HCC patients with high RAC1 expression had a shorter overall survival period. WB analysis of the clinical samples revealed that the protein concentration of RAC1 was higher in HCC tissues than in adjacent normal tissues (Fig. 6c). Immunohistochemistry staining revealed that RAC1 was more localized in the cytoplasm of adjacent non-tumor tissues, while the staining was deeper in HCC tissues (Fig. 6d).

Similarly, overexpressed RAC1 and knockdown cell lines were constructed using lentivirus vector transfection and antibiotic screening, with the corresponding protein expression shown in Fig. 6e. RAC1 overexpression could promote mitochondrial transfer, while RAC1 knockdown could reduce the transfer (Fig. 6f). Moreover, RAC1 overexpression enhanced the migration and invasion abilities of HCC cells, while their abilities were reduced upon RAC1 knockdown (Fig. 6g). Similar to these results, the tail vein lung metastasis experiment in nude mice (Fig. 6h–j) revealed that the RAC1 overexpression group exhibited stronger biological signals for the lung metastatic lesions during in vivo imaging, a relatively higher incidence of lung metastasis, and a larger area of lung metastatic lesions.

To further elucidate the specific relationship between RHOT1, RAC1, and HMGB1, we conducted a sequential regulation based on HMGB1 overexpression. The results showed that inhibiting the expression of RHOT1 and RAC1 in Lv-HMGB1 PLC cells could reduce the mitochondrial transfer, the migration and invasion ability of HCC cells, and the in vivo tail vein lung metastasis ability (Fig. 6k–o). Simultaneously, we analyzed the correlation between HMGB1, RHOT1, and RAC1 expression in tissue microarrays and clinicopathological data. RHOT1 overexpression was closely related to HBsAg positivity, while RAC1 overexpression was closely related to AFP (>20 ng/mL) (Table 1).

**Table 1:** The correlation between HMGB1, RHOT1, and RAC1 expression and clinical pathological variables in HCC patients.

	Low HMGB1 (*n* = 41)	High HMGB1 (*n* = 46)	*P-*value	Low RHOT1 (*n* = 38)	High RHOT1 (*n* = 49)	*P-*value	Low RAC1 (*n* = 40)	High RAC1 (*n* = 47)	*P-*value
Age									
≤52 (median)	18	28	0.114	18	28	0.365	18	28	0.175
å 52 (median)	23	18		20	21		22	19	
Sex									
Male	36	43	0.587	32	47	0.134	37	42	0.894
Female	5	3		6	2		3	5	
HBsAg									
Positive	31	38	0.421	26	43	0.027*	34	35	0.227
Negative	10	8		12	6		6	12	
AFP									
≤20 ng/mL	20	16	0.186	18	18	0.318	22	14	0.017*
å 20 ng/mL	21	30		20	31		18	33	
Tumor number									
single	35	41	0.598	33	43	1.000	35	41	0.970
multiple	6	5		5	6		5	6	
Maximal tumor size									
≤5 cm	26	34	0.291	26	34	0.923	28	32	0.847
å 5 cm	15	12		12	15		12	15	
TNM stage									
I–II	20	21	0.770	20	21	0.365	19	22	0.949
III–IV	21	25		18	28		21	25	
Tumor encapsulation									
Present	18	23	0.570	20	21	0.365	20	21	0.620
Absent	23	23		18	28		20	26	
GGT									
≤50 U/I	17	28	0.071	21	24	0.561	20	25	0.767
å 50 U/I	24	18		17	25		20	22	

## Discussion

Our study revealed that mitochondria can transfer from more invasive HCC cells to less invasive HCC cells, enhancing the latter’s metastasis and invasion ability. HMGB1 can promote mitochondrial transfer between HCC cells under hypoxia. Moreover, HMGB1 can promote the expression of mitochondrial transporter RHOT1 through endoplasmic reticulum stress and the expression and translocation of TNT regulatory protein RAC1. Similar to our findings, Valdebenito et al. discovered that cancer cells could improve tumor invasiveness by adapting to the microenvironment through mitochondrial metastasis [40]. Caicedo et al. observed that mitochondrial transfer from giant cells to cancer cells improved mitochondrial function, improving the proliferation and invasion abilities of these cells [41]. Wang et al. discovered that PC12 pheochromocytoma cells treated with ultraviolet light exhibited reduced apoptosis and an increased survival rate after mitochondrial transfer in co-culture with healthy cells [42]. However, Chang et al. discovered that after receiving mitochondria from healthy cells, the growth of breast cancer cells was inhibited, and their sensitivity to paclitaxel and doxorubicin was increased. However, the cell survival rate of normal human breast cell lines remained unchanged after mitochondrial transfer [41]. This indicates that the effects of intercellular mitochondrial transfer are inconsistent and require further research. It is worth thinking that intercellular mitochondrial transfer is a complex process which is crucial for the communication and energy between recipient and donor cells. Currently, the horizontal transfer of mitochondria between cells remains unexplored, and our study is also indirect. There is a lack of in vivo experiments demonstrating the direct involvement of these proteins in the mitochondrial transfer between cells. Due to the limitations of this study, our research group will continue to explore the regulation of mitochondrial transfer in relation to the deep metabolism and energy of receptor cells, as well as the direction of mitochondrial transfer.

Tunnel nanotubes (TNTs) are considered the main intercellular platforms for unidirectional and bidirectional mitochondrial exchange [9]. TNTs are long-range intercellular cytoplasmic channels for direct cell-to-cell communication; they are a type of cellular stress response that improves physiological and metabolic disorders [43]. The transported items include proteins, microRNAs, mitochondria, and even viruses [44–46]. TNTs are characterized by membrane channels containing F-actin, composed of various isomers, including α-actin, β-actin, and γ-actin. Polymerization of actin is crucial for the formation of TNTs, and its depolymerization drugs can inhibit the formation of TNTs [47]. Structurally, the width of TNTs ranges from 50–1000 nm, and the length ranges from a few to 100 μm [48]. Few studies have reported TNT formation in cell lines of bladder cancer, urothelial cancer, breast cancer, cervical cancer, colon cancer, mesothelioma, osteosarcoma, ovarian cancer, adenocarcinoma, pheochromocytoma, prostate cancer, and squamous cell carcinoma [49]. TNTs-mediated mitochondrial translocation alters energy metabolism in recipient cells, including the increased production of OXPHOS and ATP in various cellular systems [50–52]. Several studies have shown that the transferred mitochondria can exert their effect within a minimum of 45 cell passages (135 days) in vitro [53], while other studies discovered maintenance of acquired phenotype for at least 21 days [54]. The three common inducers of TNT formation typically work together: chemical or physical stressors, inflammatory conditions, and metabolic stress [55]. TNT formation can be promoted by hypoxia in various cancers, including ovarian and colon cancer [36]. As a solid tumor, HCC is characterized by hypoxia in its microenvironment. Therefore, the increase in TNT formation under hypoxic conditions in HCC may be one of the reasons for the increased mitochondrial transfer between HCC cells in our experiments after hypoxia [56]. Accumulating evidence indicates that TNTs play a role in connecting cells in cancer microenvironments, and their structural components have become potential targets for drug therapy or other targeted strategies [36]. In the breast cancer model, the chemical inhibitor of the Ras/Rho GTPase signal inhibited TNT formation and improved the efficacy of immunocheckpoint inhibitor [24]. However, TNT-mediated mitochondrial transfer may be associated with chemical resistance and play a role in acquired cancer cell resistance [57]. This indicates that TNTs and TNT-mediated mitochondrial transfer have great potential for future cancer treatments.

RHOT1, a key protein in mitochondrial transport, can regulate the spatial localization of mitochondria within and between cells [58]. Currently, the role of RHOT1 in mitochondrial localization is mostly studied in neurons, and its involvement in cancer requires further research. Several studies have shown that the expression of RHOT1 is significantly increased in pancreatic cancer patients; siRNA-RHOT1 significantly inhibits the migration and invasion of SW1990 pancreatic cancer cells, with the mechanism mainly involving the regulation of SMAD4 expression [31]. In our study, the expression of RHOT1 was relatively increased in HCC patients; HCC patients with high expression exhibited relatively shorter survival periods; RHOT1 overexpression not only promoted mitochondrial transfer but also promoted metastasis and invasion of HCC cells.

RAC1, a cytoskeleton regulatory protein, is involved in extracellular signal transduction to the actin cytoskeleton and other functions. Based on other standard components of cell actin, RAC1 mediates TNT biogenesis through ARP2/3 nucleation and actin polymerization [59]. Our results indicate that RAC1 expression increased after hypoxia in HCC cells and a phenomenon of migration leading to cell membrane aggregation, which may be closely associated with the promotion of TNT formation. Moreover, the role of RAC1 in cancer cell proliferation, migration, and drug resistance has been extensively documented in many cancer studies. RAC1 is not only involved in the tumor cell cycle, proliferation, migration, invasion, and angiogenesis but is also involved in the regulation of cancer stem cells and immune evasion mediated by tumor microenvironment [38, 60]. RAC1 is overexpressed in different types of tumors, including colorectal and gastric cancers, and is associated with a poor prognosis [60]. RAC1 is considered a potential target for combating cancer and various diseases like inflammation, metabolism, and neurodegeneration. The current research explores the surprising application prospects of RAC1 inhibitors in cancer prevention and treatment, especially in HCC [61–63]. It is considered an effective target for limiting the acquisition and intrinsic resistance of HCC tumors [64]. Compounds such as GYS32661 and MBQ-167, which are currently in the preclinical development phase, can be combined with chemotherapy and/or immunotherapy targets in an innovative and promising manner [61, 62]. Moreover, our study revealed that inhibiting RAC1 expression in HCC cells could reduce mitochondrial transfer between HCC cells, and the migration and invasion abilities of HCC cells were significantly reduced.

HMGB1 can induce endoplasmic reticulum stress through TLR4/RAGE and other pathways [16, 65]. Moreover, hypoxia can induce endoplasmic reticulum stress, and its perception and response are coordinated by the unfolded protein response (UPR) [63]. Several studies have shown that multiple regulatory elements involved in crosstalk between the UPR and mitochondria play a role in mitochondrial dynamics, bioenergetics, and mitochondrial autophagy [66]. Several studies have shown that when the UPR is activated, ATF6α and ATF6β can form heterologous protein complexes with the NF-Y complex [35]. The NF-Y complex is a unique trimeric transcription factor formed by the histone folding domain (HFD) NFYB/NFYC subunit and NFYA. All subunits were overexpressed in the HCC TCGA database [67]. NFYC subunit promotes tumor growth in glioma [68] and functions as an oncogene in choroid plexus carcinoma [69]. Our study revealed that the expression of NFYA and NFYC increased after hypoxia and HMGB1 stimulation, which can serve as transcription factors to enhance the expression of RHOT1. Moreover, we discovered that HMGB1 could promote RAC1 expression and regulate its translocation under hypoxia, which may be beneficial for the formation of intercellular TNTs under hypoxia.

In conclusion, we confirmed mitochondrial transfer between HCC cells via TNTs. The transfer of mitochondria from highly invasive HCC cells to less invasive HCC cells can improve the migration and invasion ability of the latter. Hypoxic conditions increased mitochondrial transfer between HCC cells. RHOT1, a mitochondrial transport protein, promotes mitochondrial transfer and migration as well as metastasis of HCC cells during this process. Under hypoxia, HMGB1 can further regulate RHOT1 expression by increasing the expression of the NFYA and NFYC subunits in the NF-Y complex. RAC1, the protein associated with the formation of TNTs, promotes mitochondrial transfer and the migration and invasion of HCC cells. Moreover, HMGB1 can regulate RAC1 aggregation to the cell membrane under hypoxia. HCC patients with high HMGB1, RHOT1, or RAC1 expression exhibited a relatively shorter overall survival period.

### Abbreviations

HCC: Hepatocellular carcinoma; TNTs: tunneling nanotubes; DMEM: dulbecco modified eagle medium; FBS: fetal bovine serum; CoIP: Co-immunoprecipitation; ChIP: Chromatin immunoprecipitation; WB: Western blot; qRT-PCR: Quantitative real-time polymerase chain reaction; FESEM: field emission scanning electron microscope; IF: immunofluorescence; GFP: green fluorescence protein; RFP: red fluorescence protein; FACS: fluorescence-activated cell sorting; HMGB1: high-mobility group protein 1; TM: tunicamycin; UPR: unfolded protein response; PERK: protein kinase-like ER kinase; ATF6: activated transcription factor 6; IRE1: inositol required enzyme 1; EP: ethyl pyruvate.

### Supplementary information

Supplementary figure and information

checklist

Original western blots

## Data Availability

The data generated and analyzed in this study are available from the corresponding authors upon request.
