# Supplementary material for: HMGB1 promotes mitochondrial transfer between hepatocellular carcinoma cells through RHOT1 and RAC1 under hypoxia
Source: Cell Death Dis. 2024 Feb 20;15(2):155. doi: 10.1038/s41419-024-06536-6 (PMC10879213; doi:10.1038/s41419-024-06536-6)
Supplement: Supplementary file 1 — Supplementary figure and information [file 41419_2024_6536_MOESM1_ESM.pdf]

SUPPLEMENTAL FIGURE

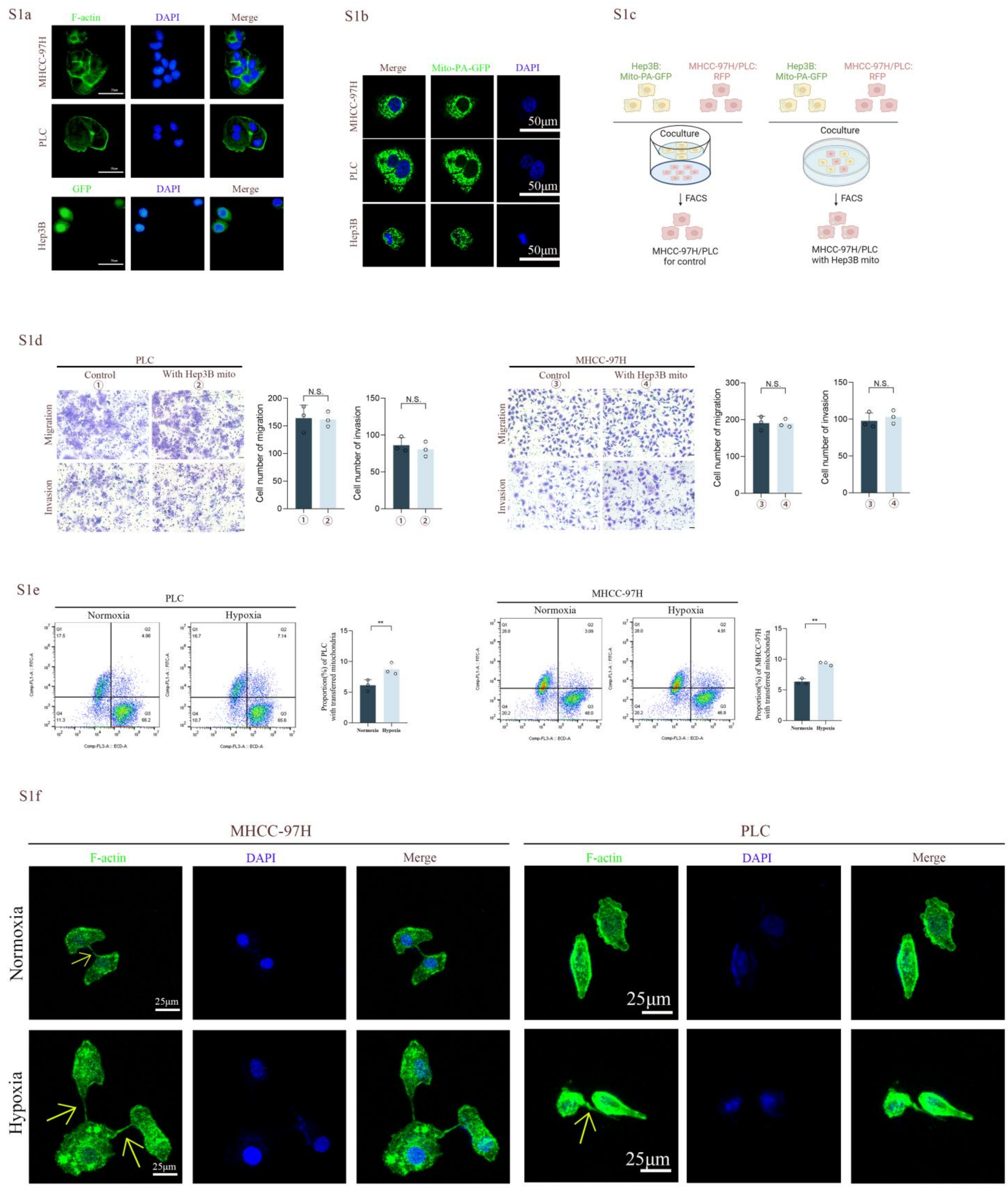

S1a Immunofluorescence images showed phalloidin F-actin staining (green) in MHCC-97H and PLC cells, GFP (green) in Hep3B. Scale bars: 20 μm

S1b Immunofluorescence staining showed mitochondria (green) in MHCC-97H and PLC cells labeled with Mito-PA-GFP. Scale bars: 50 μm

S1c-d The schematic diagram shows MHCC-97H and PLC cells labeled with RFP, co cultured with Hep3B cells labeled with Mito-PA-GFP, and then subjected to sterile cell flow cytometry sorting. After sterile cell flow cytometry sorting, they were subjected to metastasis and invasion experiments. Scale bars: 50μm. n =3. N.S. no significance.

S1e Hep3B cells labeled with Mito-PA-GFP were co-cultured with MHCC-97H and PLC cells labeled with RFP under normoxic and hypoxic conditions for 24 h, and flow cytometry was performed to determine the proportion of MHCC-97H and PLC cells containing transferred mitochondria. n =3, **P < 0.01.

S1f Confocal results showed TNT (yellow arrow) between HCC cells under normoxic and hypoxic conditions. Scale bars: 25 μm.

S2a

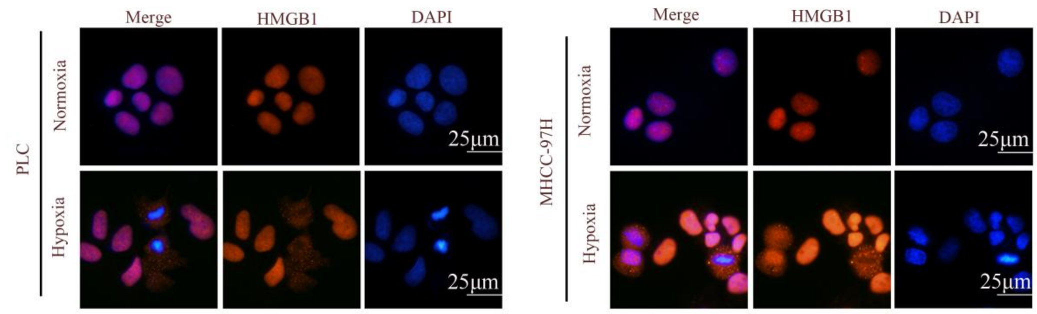

S2b

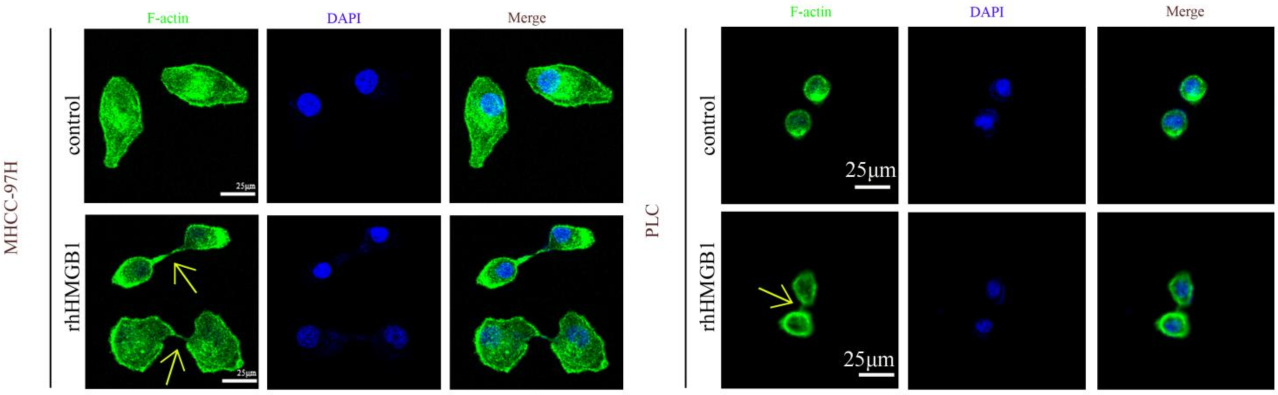

S2c

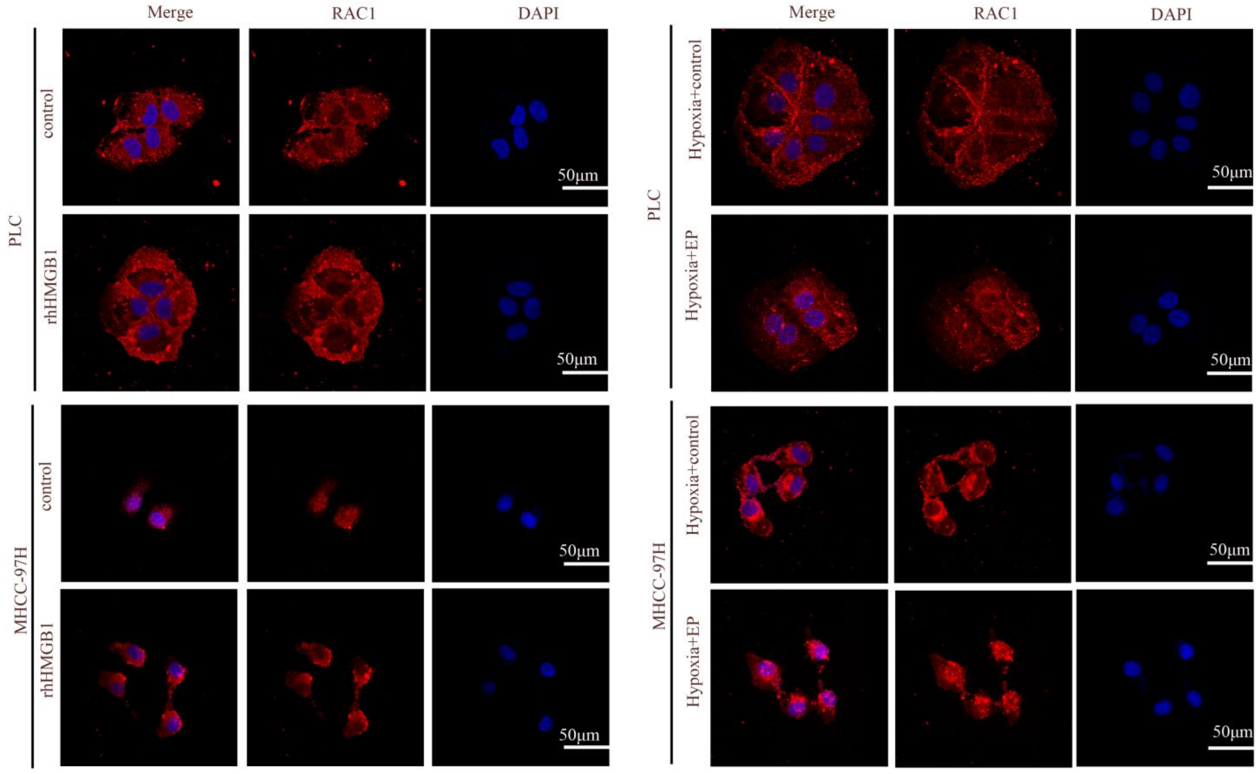

S2d

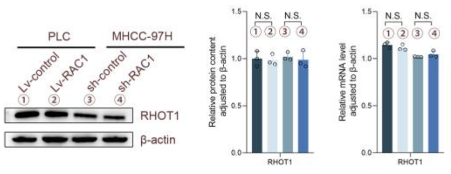

S2a Immunofluorescence staining showed the localization of HMGB1 in PLC and MHCC-97H before and after hypoxia. Scale bars: 25 μm.

S2b Confocal results showed TNT (yellow arrow) between HCC cells after stimulation with recombinant

human HMGB1 protein. Scale bars: 25 μm.

S2c Immunofluorescence staining showed the localization of RAC1 in PLC and MHCC-97H after stimulation with recombinant human HMGB1 protein and EP treatment under hypoxic conditions. Scale bars: 50 μm.

S2d Protein content and mRNA levels of RHOT1 after RAC1 lentivirus transfection into PLC and MHCC-97H cells. n =3. N.S. no significance.

SUPPLEMENTAL INFORMATION

1、Reagents and antibody

Reagent	Company	Catalog
MitoTracker-Red	Cell Signaling Technology	9082
ActinGreen	KeyGEN BioTECH	KGMP001
Cytochalasin D	Apexbio	B6645
Recombinant human HMGB1 protein	Sigma-Aldrich	SRP6265
Tunicamycin	abcam	11089-65-9
4-Phenylbutyric acid	MedChemExperss	HY-A0281
Ethyl pyruvate	MedChemExperss	617-35-6
Matrigel	Corning	354277
Antibody		
β-actin	Proteintech	60008-1-lg
HMGB1	ABclonal	A19529
RHOT1	ABclonal	A5838
RHOT1 for IP	Santa Cruz Biotechnology	sc-398520
GRP78	ABclonal	A0241
IRE1	ABclonal	A17940
PERK	ABclonal	A18196
XBP1	ABclonal	A1731
ATF6	ABclonal	A0202
ATF6 for IP	Proteintech	24169-1-AP
NFYA	Santa Cruz Biotechnology	Sc-17753
NFYB	Santa Cruz Biotechnology	Sc-376546
NFYC	Santa Cruz Biotechnology	Sc-390861
RAC1	Proteintech	66122-1-lg
Arp2	ABclonal	A5734
Arp3	ABclonal	A4514
NA^+/K^+-ATPase	Proteintech	14418-1-AP

2、Sequences used in the study

shRNA	
shHMGB1	CCGGGGAGAGATGTGGAATAACACTCGAGTG TTATTCCACATCTCTCCTTTTTG
shRHOT1	CCGGGAAGAAAATTCGTGAAGATCTCGAGATC TTCACGAATTTTCTTCTTTTTG
shRAC1	CCGGCCTTGTGAGTCCTGCATCACTCGAGTGA TGCAGGACTCACAAGGTTTTTG

siRNA	
siXBP1	5’-TGAGAACCAGGAGTTAAGA -3’
siPERK	5’-GGAAACAGCTATTCTCATA -3’
siATF6	5’-GCAACCAATTATCAGTTTA -3’
Primers for real-time PCR:	
β-actin sense:	5’- CATGTACGTTGCTATCCAGGC -3’
β-actin antisense:	5’- CTCCTTAATGTCACGCACGAT -3’
RHOT1 sense:	5'-AAGGTAACAAGTCGATGGATTCC-3’
RHOT1 antisense:	5'-TCAGGTTTTTCGCTGAACACT-3’
NFYA sense:	5’- CAGACTCAGACACAGCAGCAGATTG -3’
NFYA antisense:	5’- GGGATTCTTTGGATAGCAGGCACAG -3’
NFYB sense:	5’- GCCATACCTCAAACGGGAAAGATTG -3’
NFYB antisense:	5’- GCTGTGACTGCTCCACCAATTCC -3’
NFYC sense:	5’- AGGTTGGAGAAGGTCAGCAGGTG -3’
NFYC antisense:	5’- TGAGCATTGGTGGCAAGTGTCTG -3’
Primers used for ChIP	
binding site 1 sense:	5'- GGTCTGGTGAGAAGATCC- 3'
binding site 1 antisense:	5'- GGAATTTGAATGGCGGATT- 3'
binding site 2 sense:	5'- CCCTACTCGATCAAGCATT- 3'
binding site 2 antisense:	5'- ATCTTCTCACCAGACCATAA- 3'
binding site 3 sense:	5'- ACGCTGGGTAAGCCTGAGTAAA- 3'
binding site 3 antisense:	5'- AGGATCCGGCGCTTTCCACT- 3'
distant region sense:	5’-AGACTGGCAGAAGTATAGATG-3’
distant region antisense:	5’-AGTGAGCAATGGATATGGTT-3’
