# Supplementary material for: HMGB1 promotes mitochondrial transfer between hepatocellular carcinoma cells through RHOT1 and RAC1 under hypoxia
Source: Cell Death Dis. 2024 Feb 20;15(2):155. doi: 10.1038/s41419-024-06536-6 (PMC10879213; doi:10.1038/s41419-024-06536-6)
Supplement: Supplementary file 3 — Original western blots [file 41419_2024_6536_MOESM3_ESM.ppt]

## Slide 1
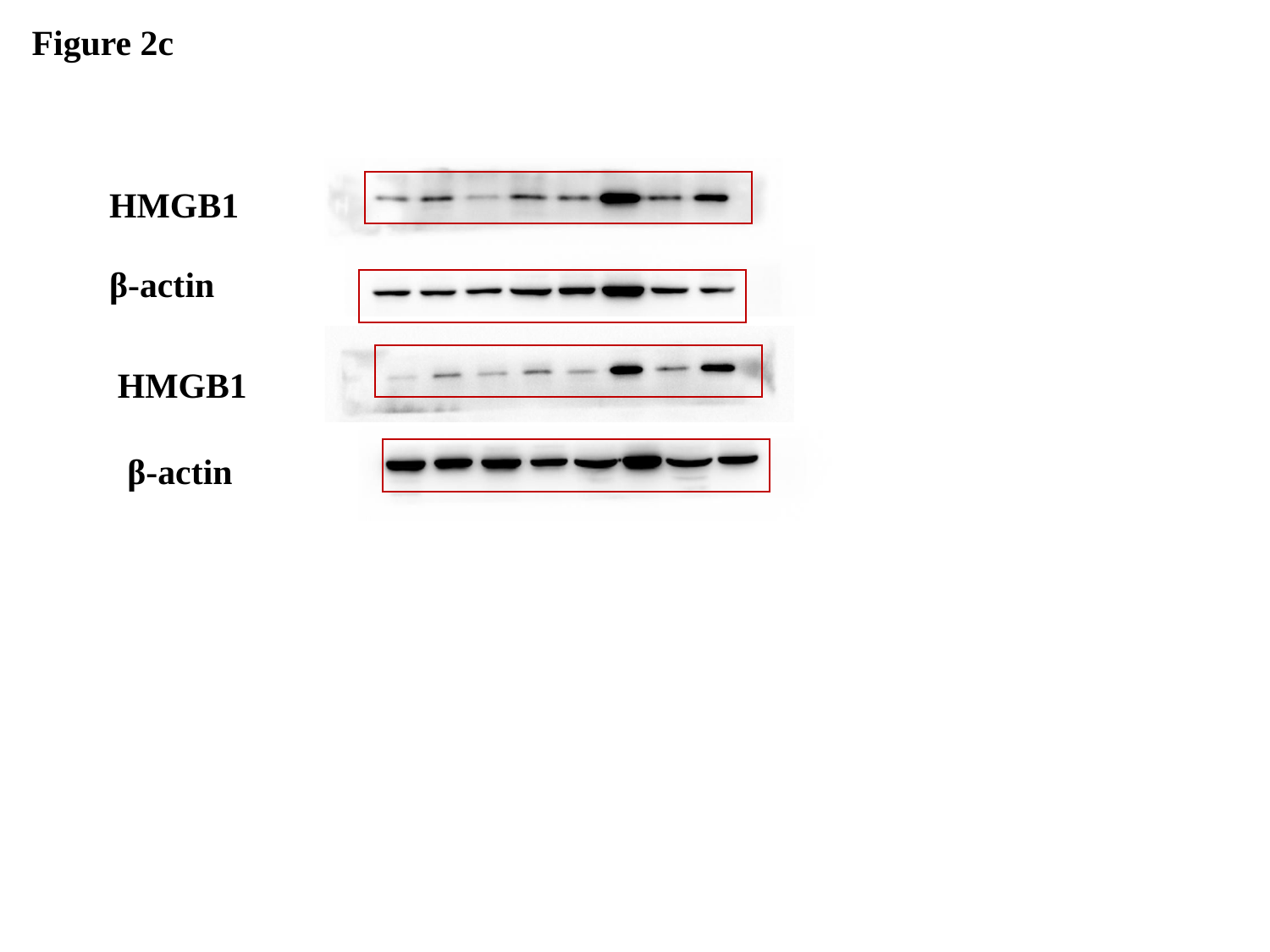

Figure 2c
HMGB1
β-actin
HMGB1
β-actin

## Slide 2
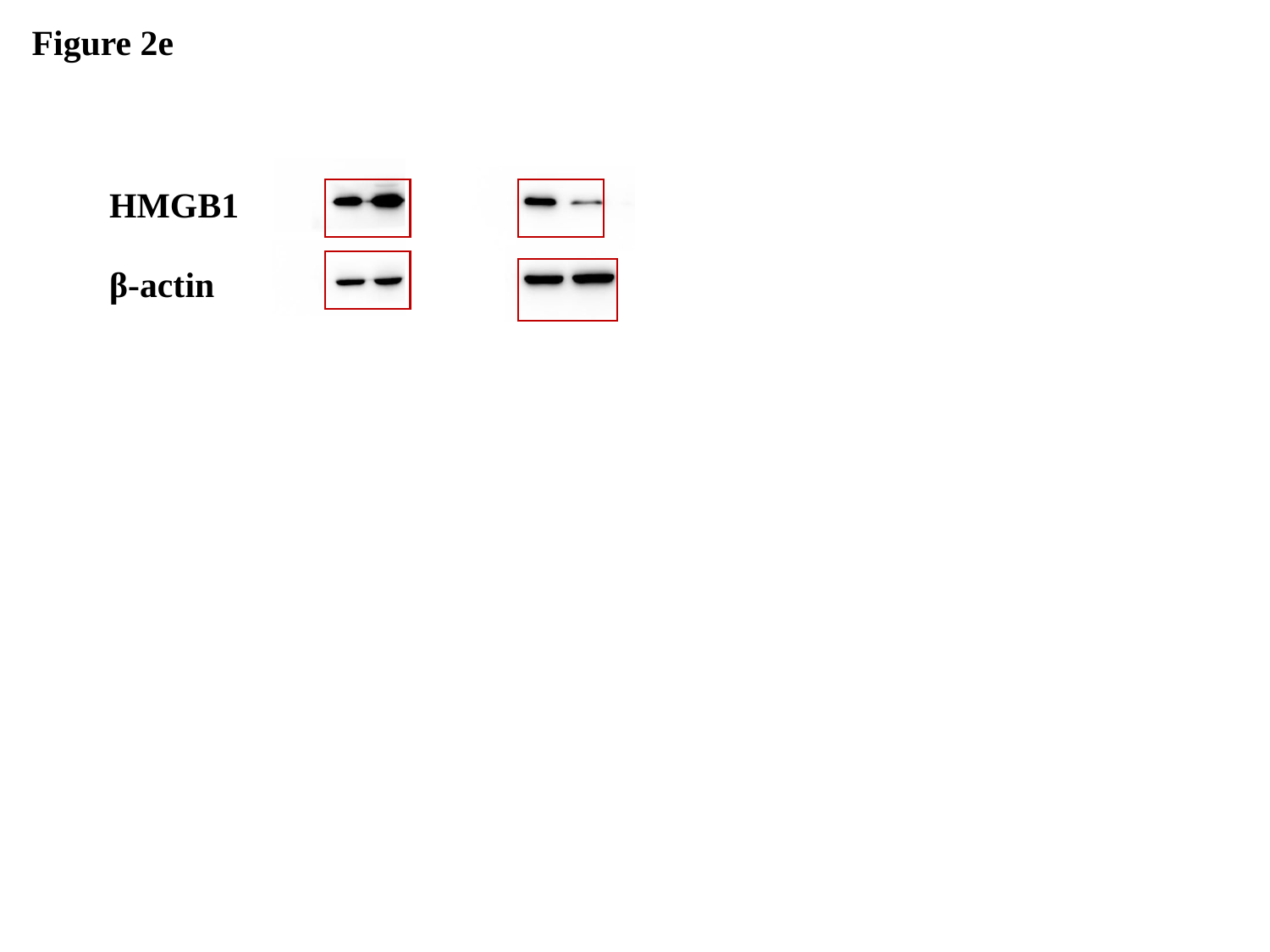

Figure 2e
HMGB1
β-actin

## Slide 3
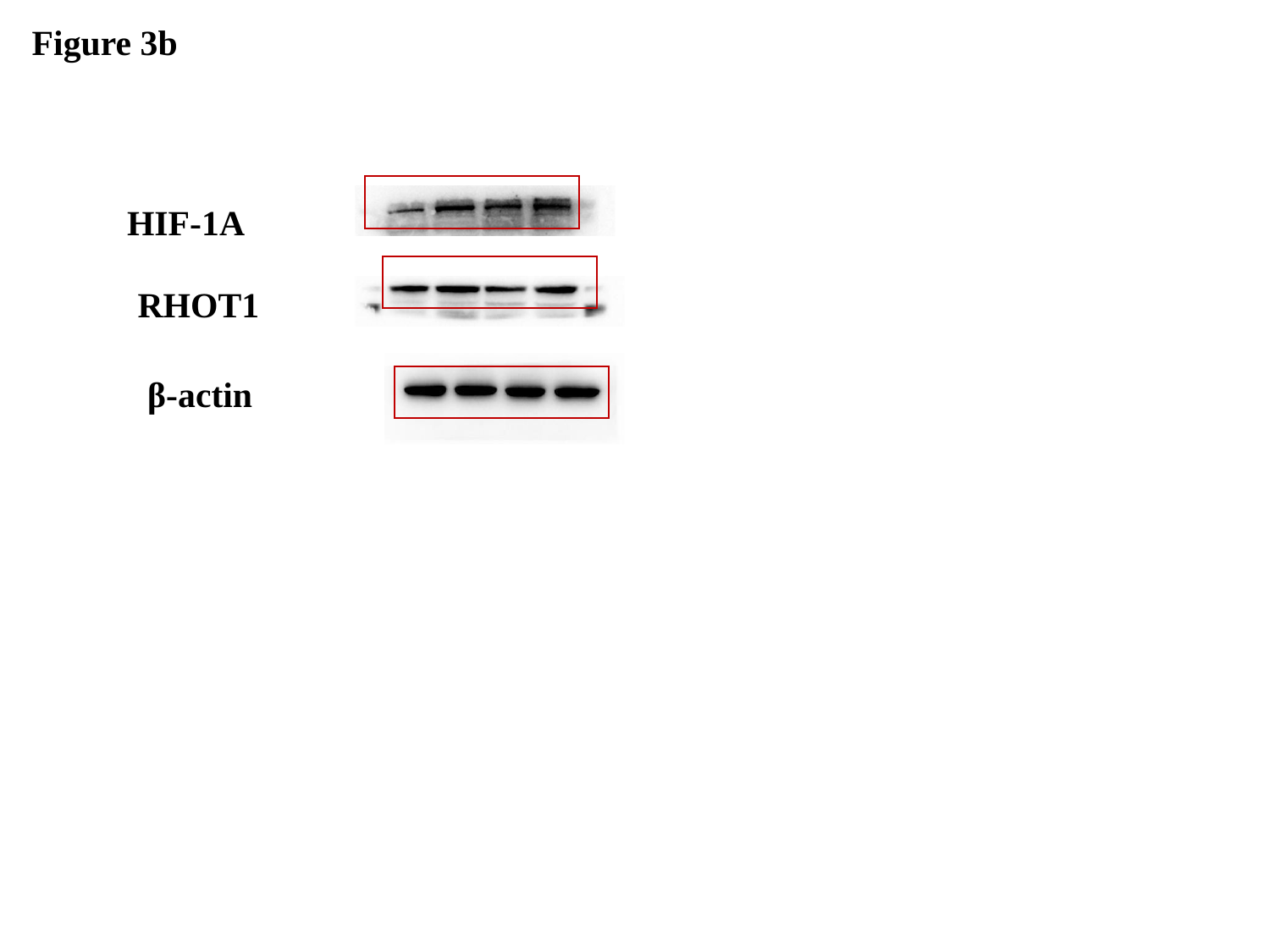

Figure 3b
HIF-1A
RHOT1
β-actin

## Slide 4
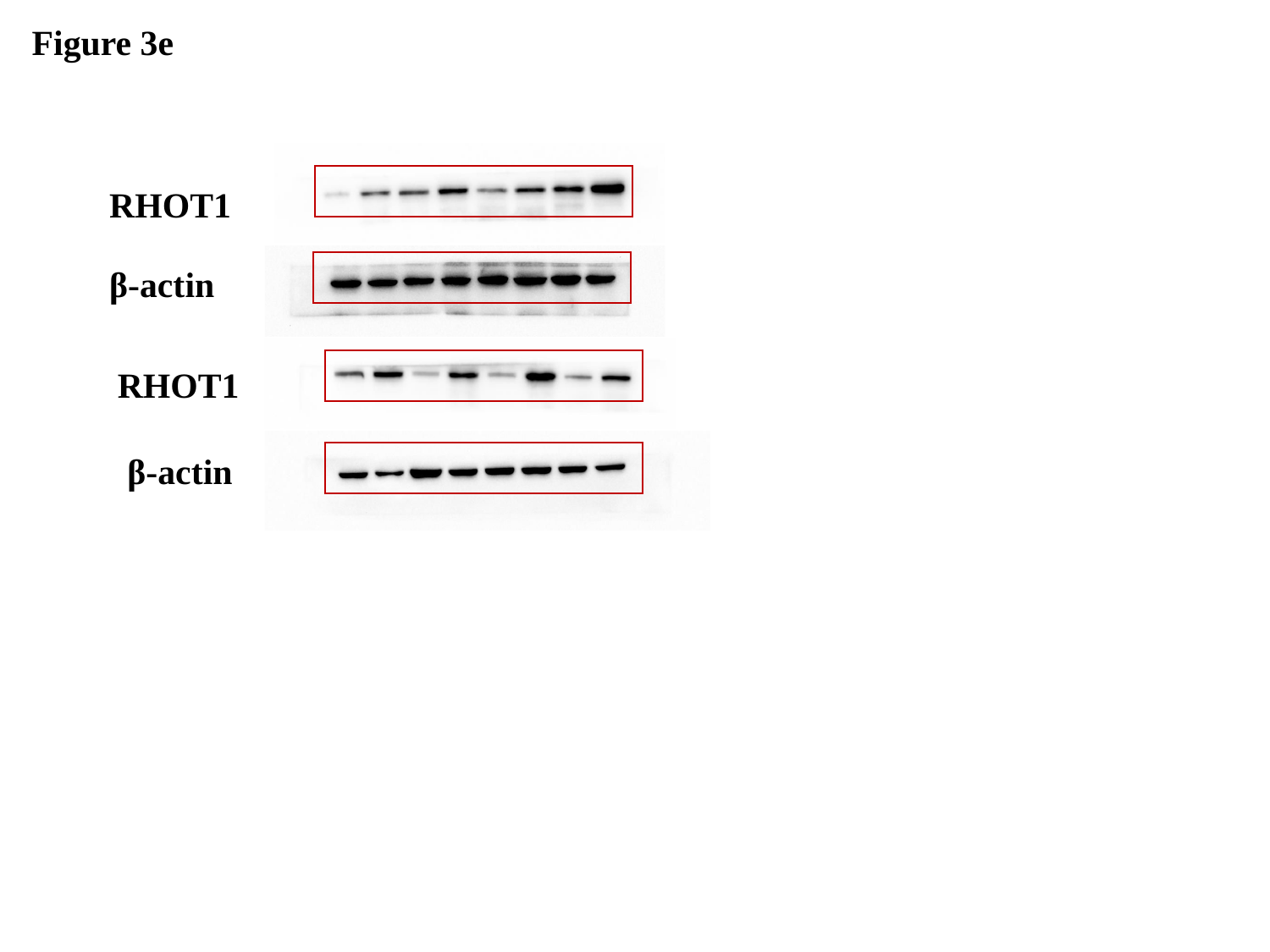

Figure 3e
RHOT1
β-actin
RHOT1
β-actin

## Slide 5
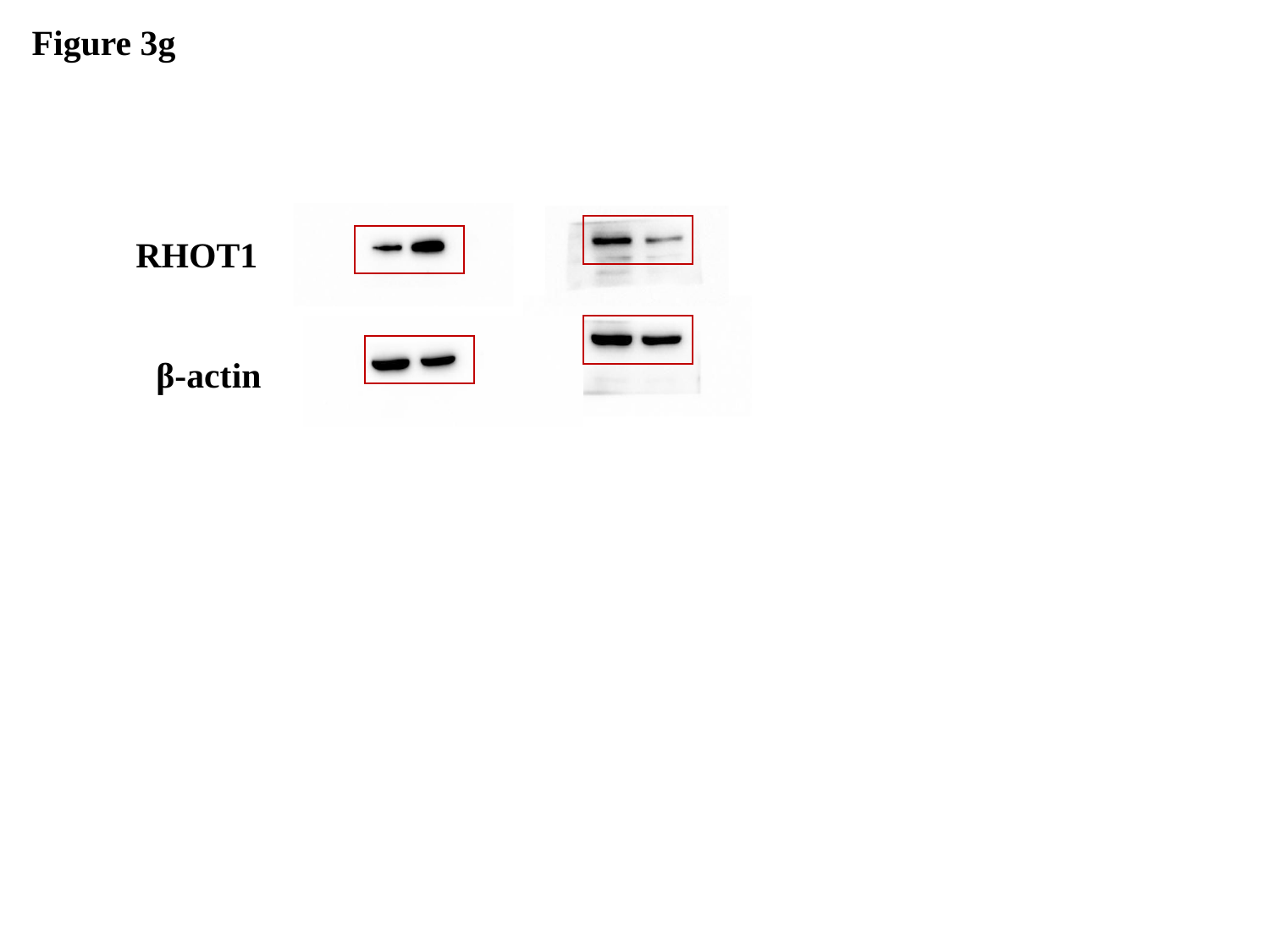

Figure 3g
RHOT1
β-actin

## Slide 6
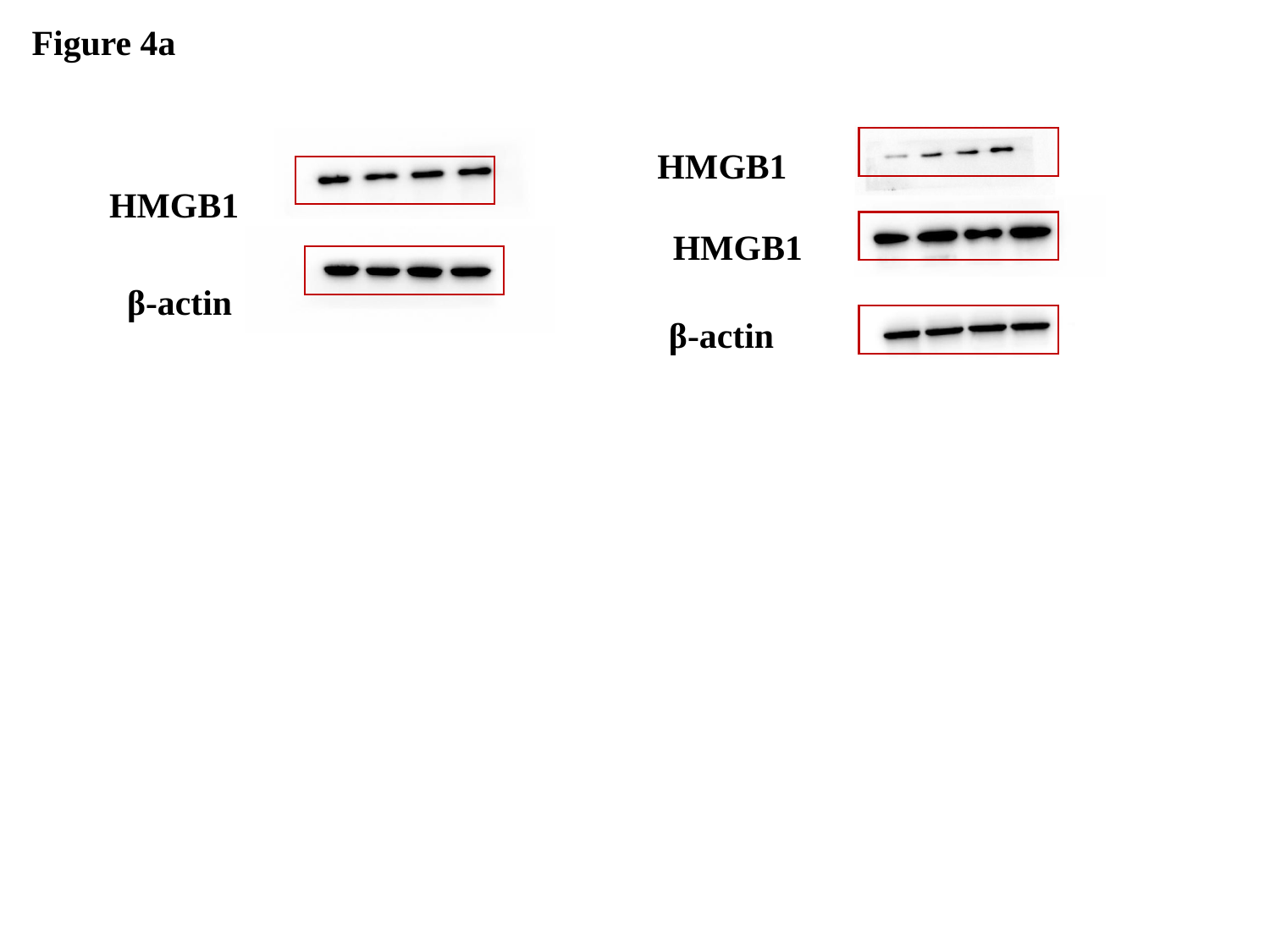

Figure 4a
HMGB1
HMGB1
HMGB1
β-actin
β-actin

## Slide 7
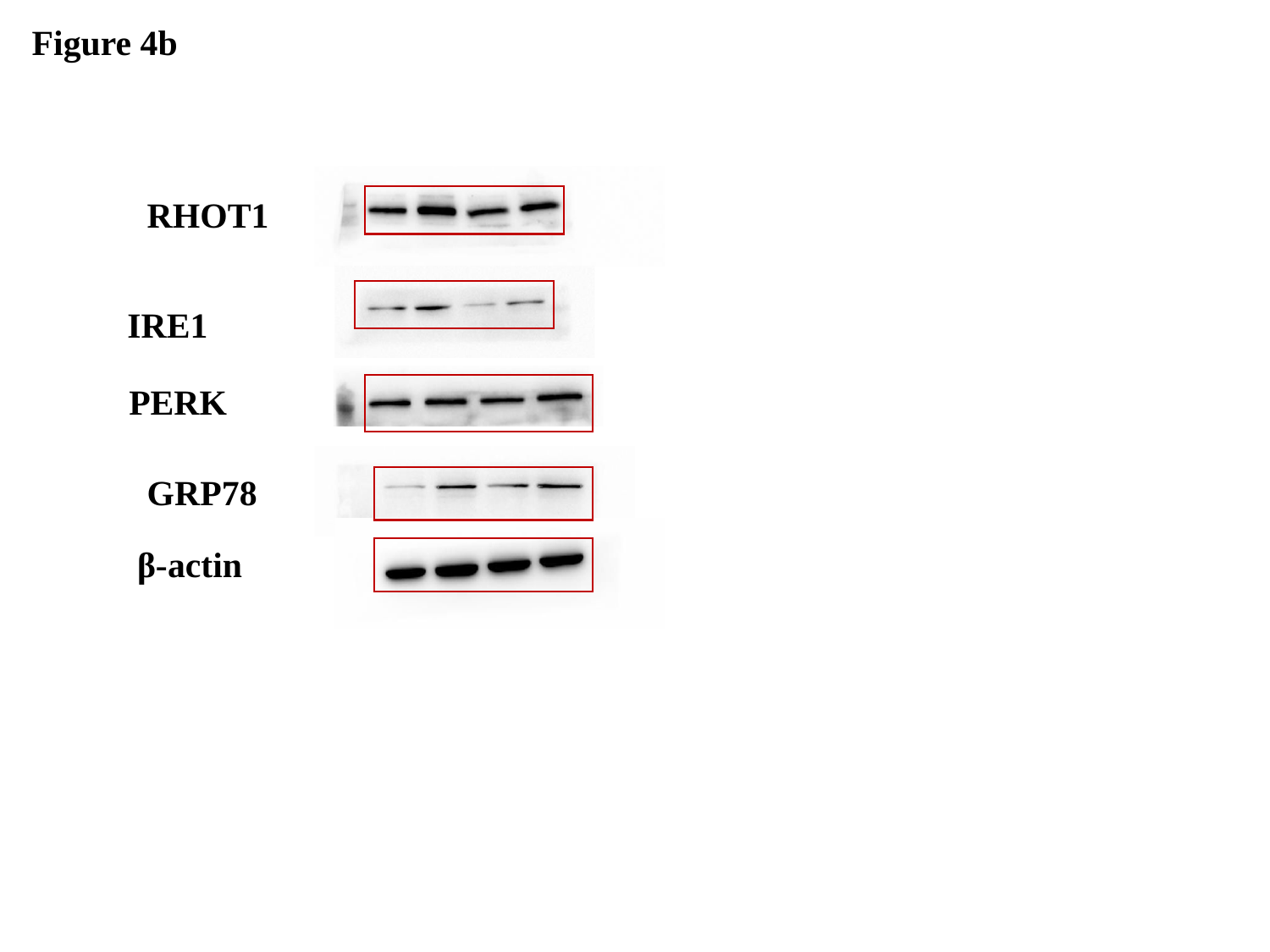

Figure 4b
RHOT1
IRE1
PERK
GRP78
β-actin

## Slide 8
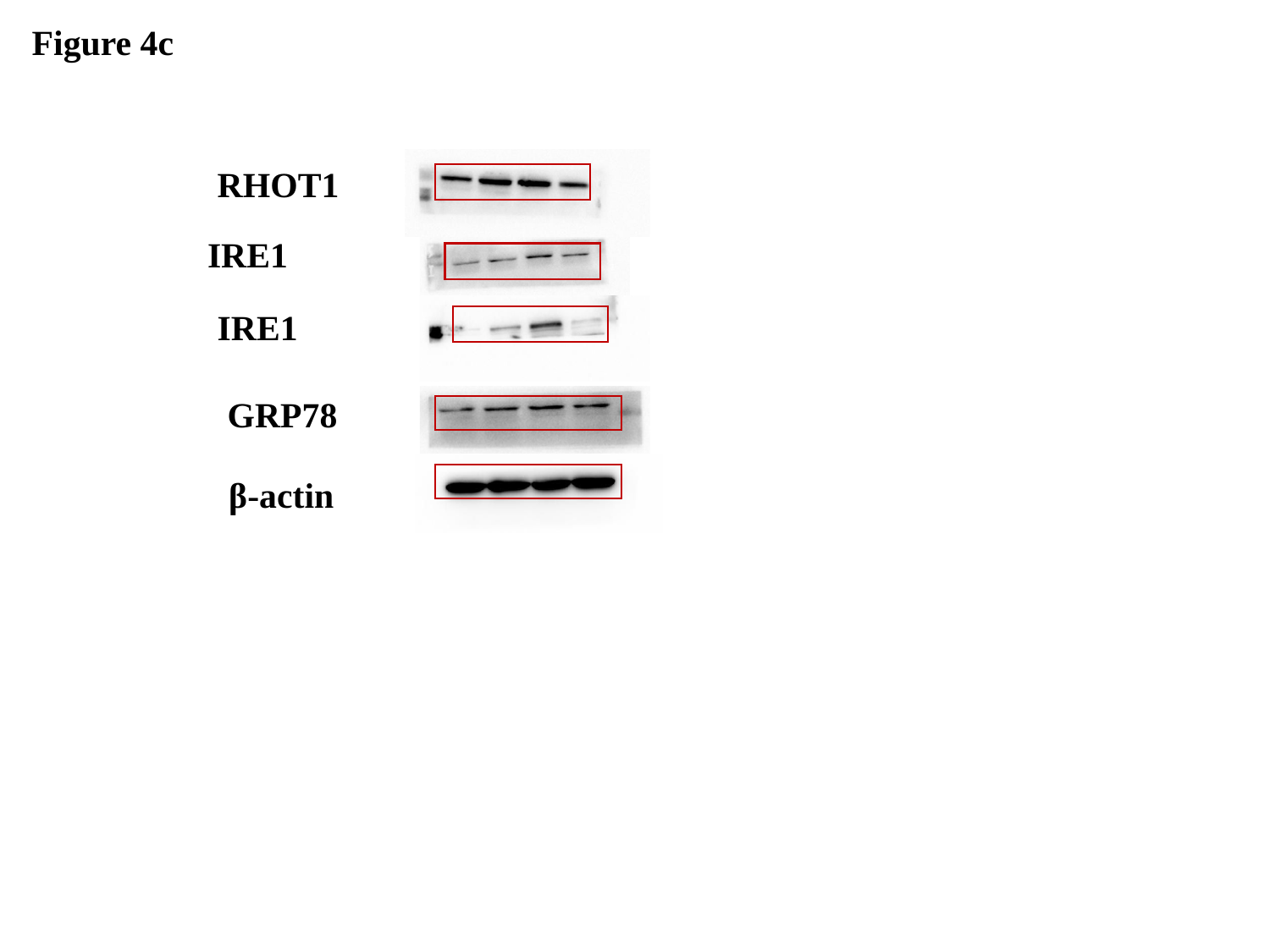

Figure 4c
RHOT1
IRE1
IRE1
GRP78
β-actin

## Slide 9
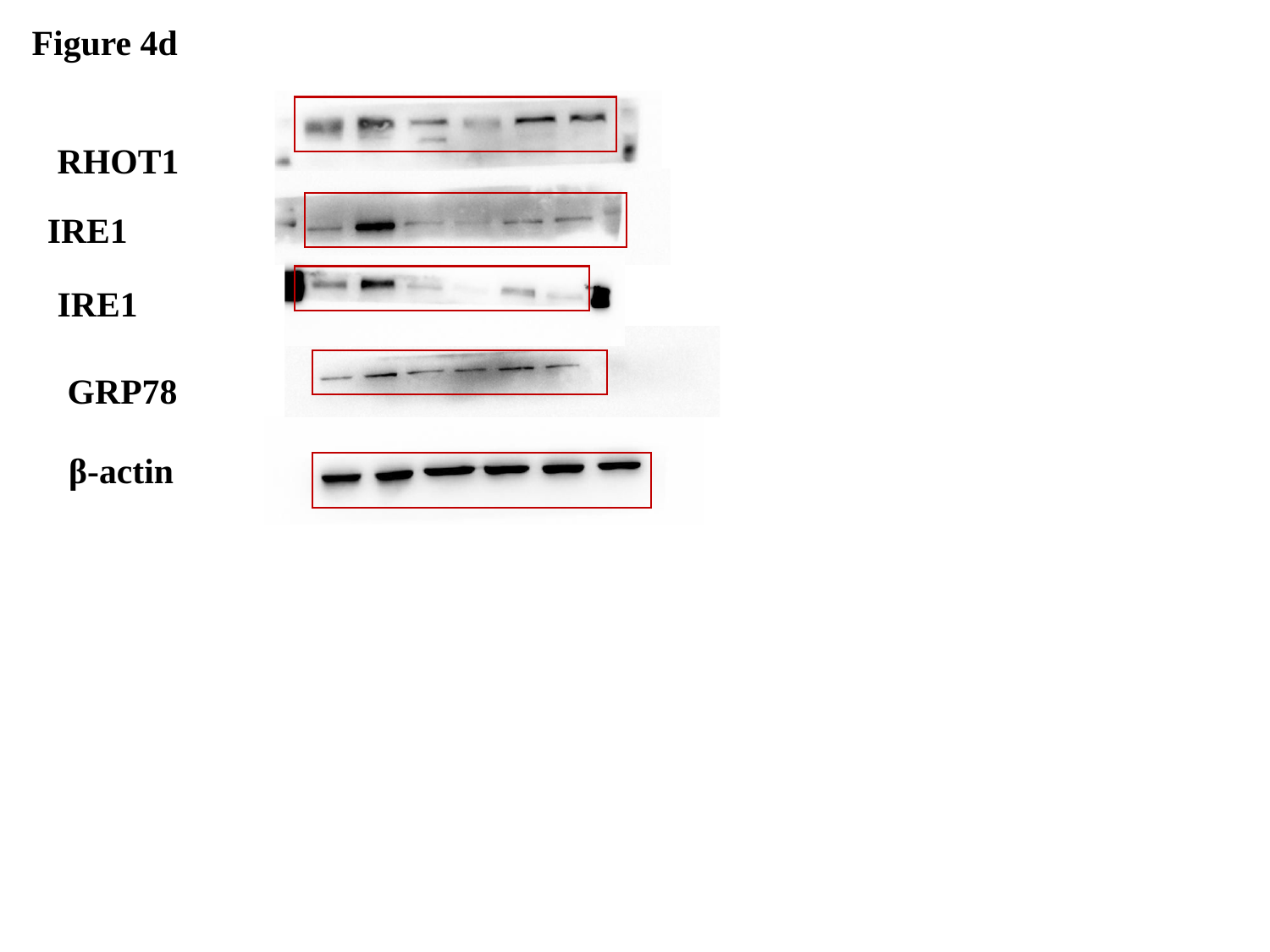

Figure 4d
RHOT1
IRE1
IRE1
GRP78
β-actin

## Slide 10
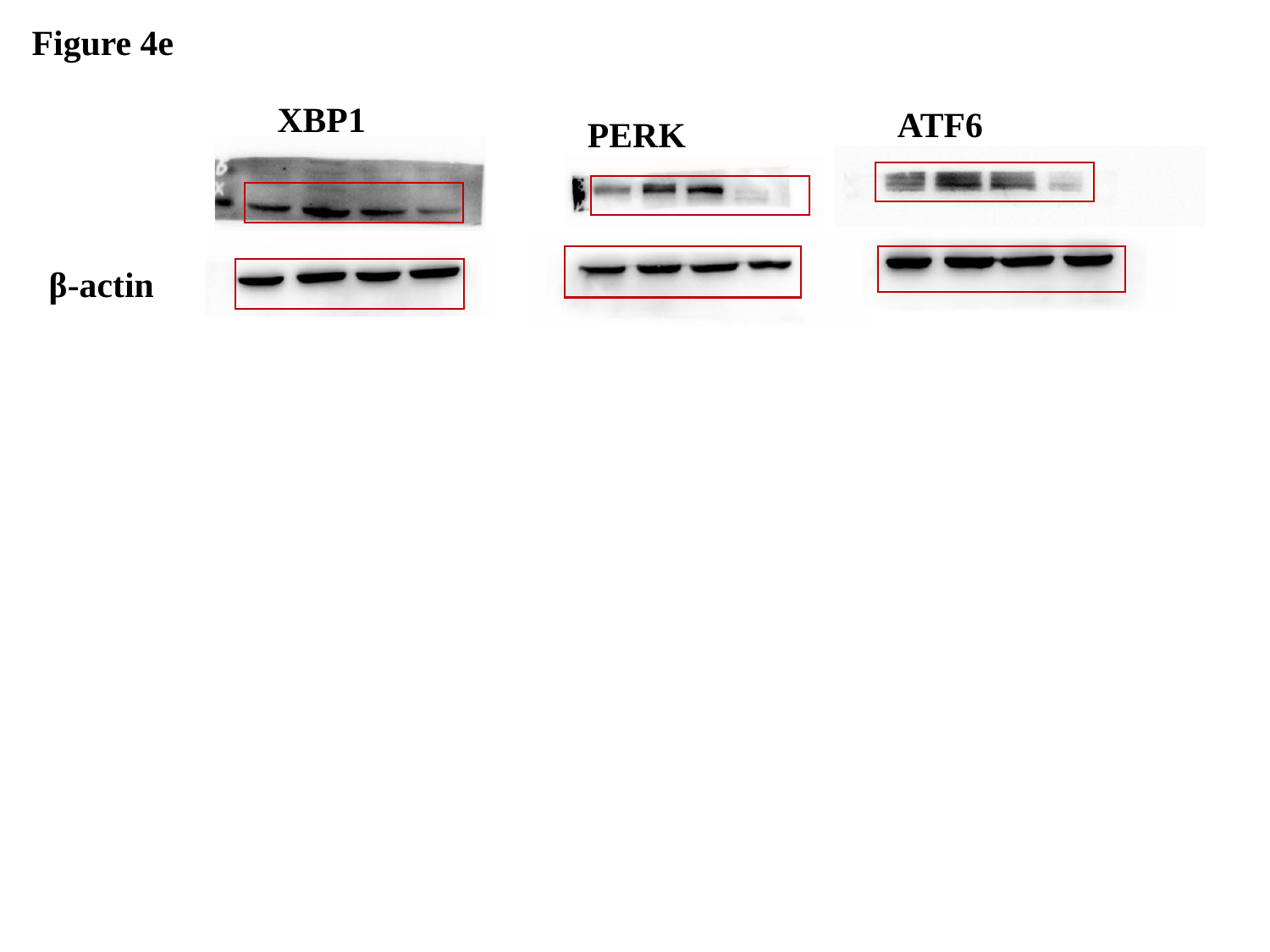

Figure 4e
XBP1
ATF6
PERK
β-actin

## Slide 11
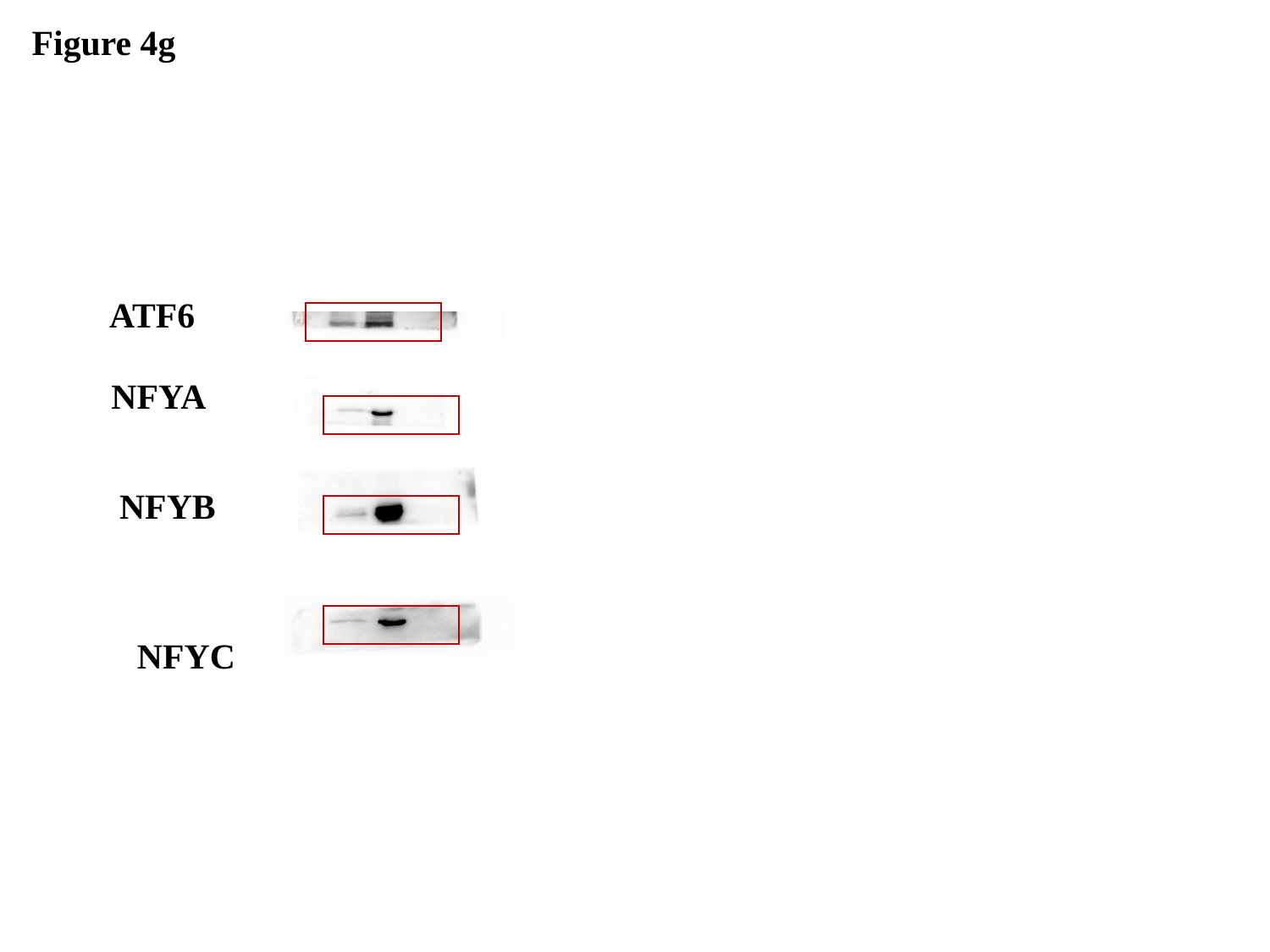

Figure 4g
ATF6
NFYA
NFYB
NFYC

## Slide 12
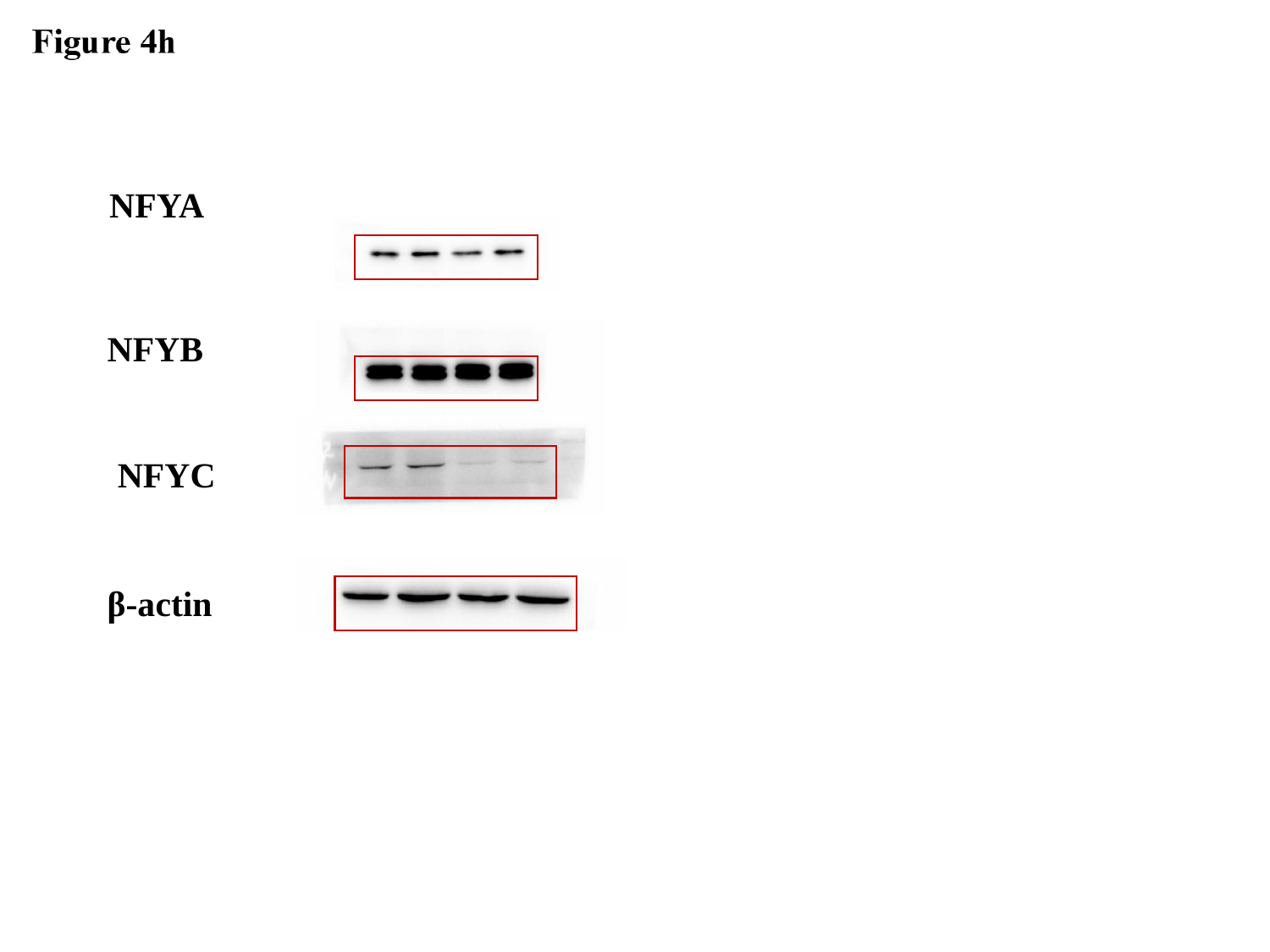

NFYA
NFYB
NFYC
β-actin

## Slide 13
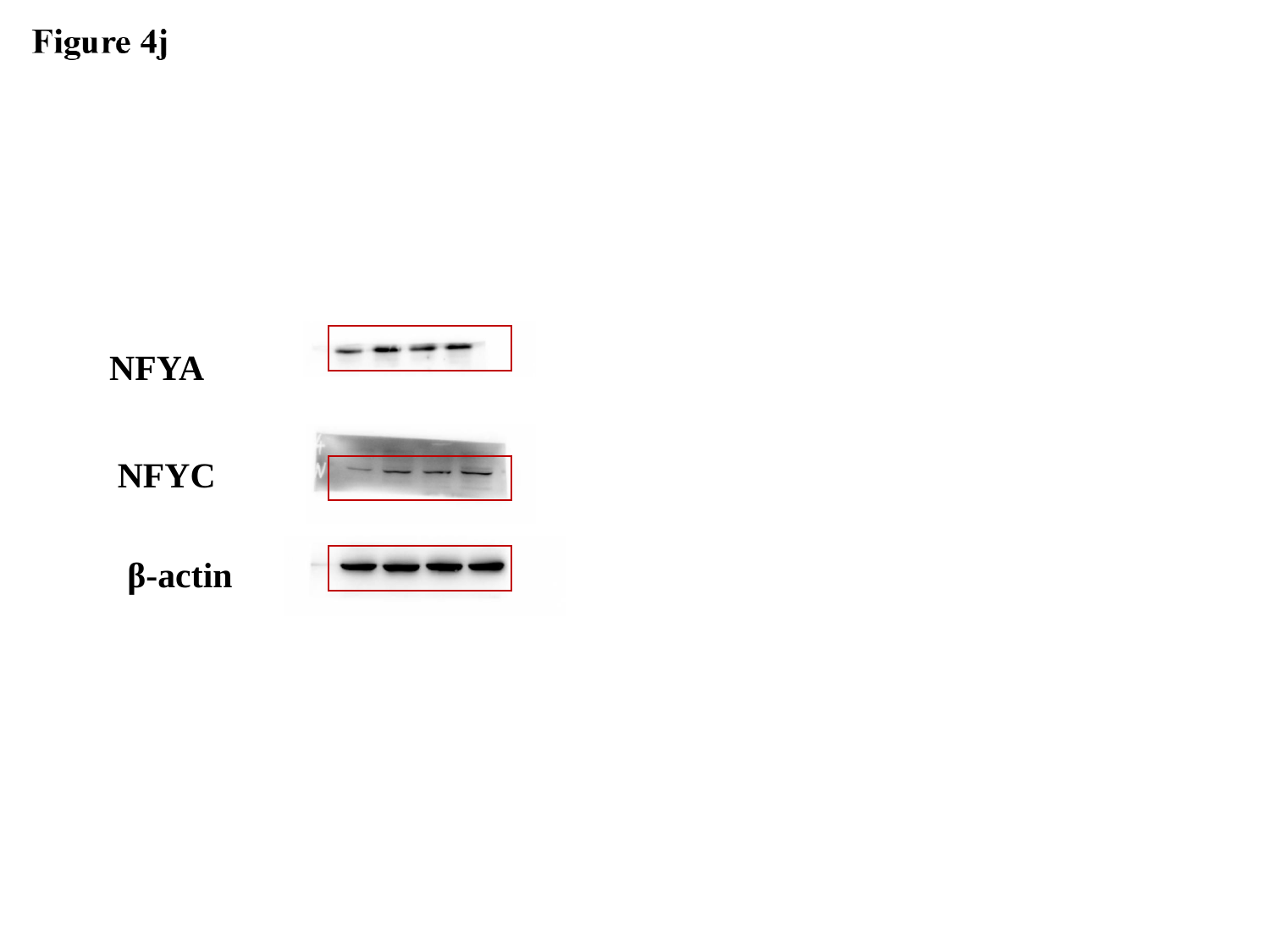

NFYA
NFYC
β-actin

## Slide 14
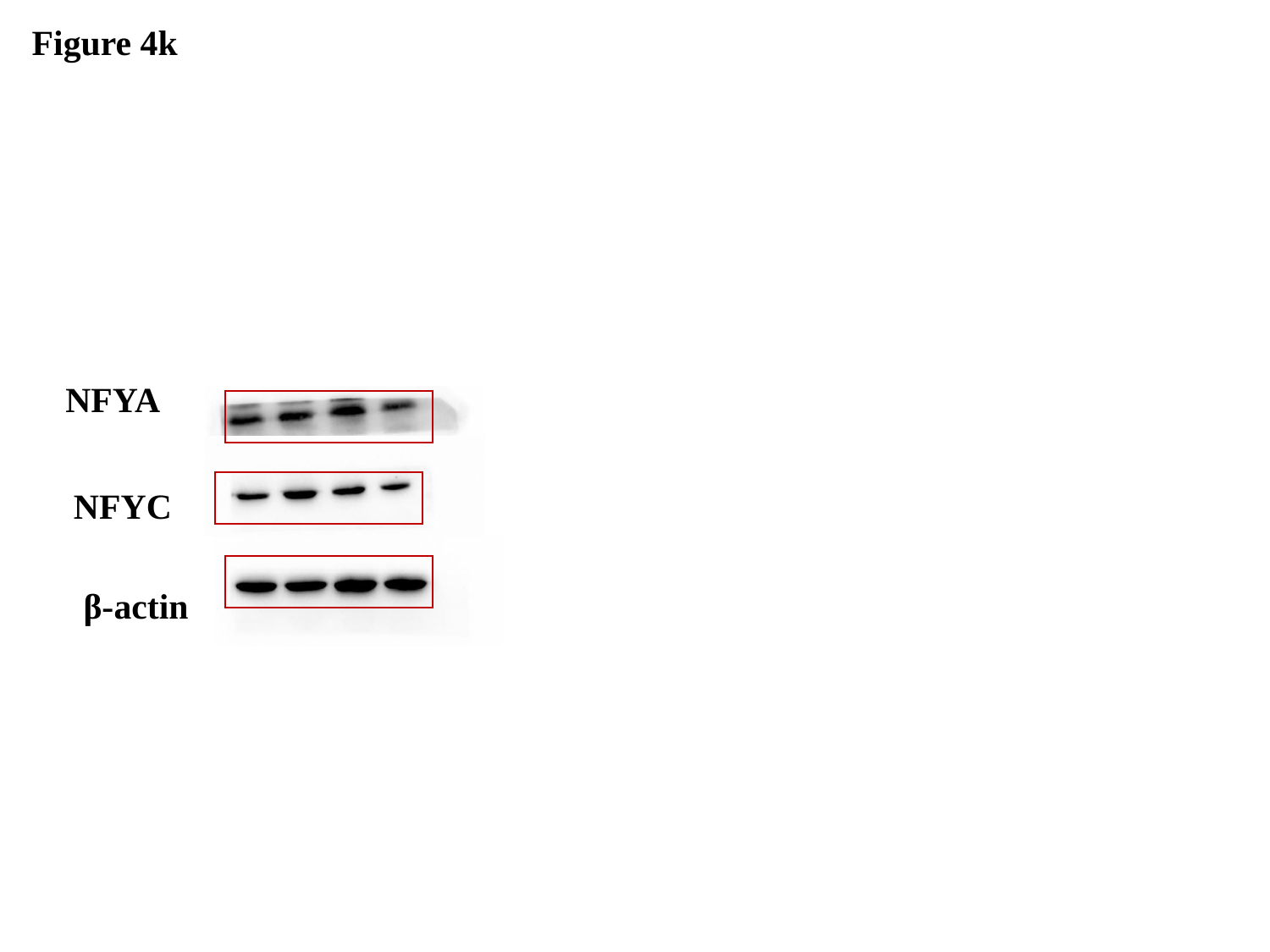

Figure 4k
NFYA
NFYC
β-actin

## Slide 15
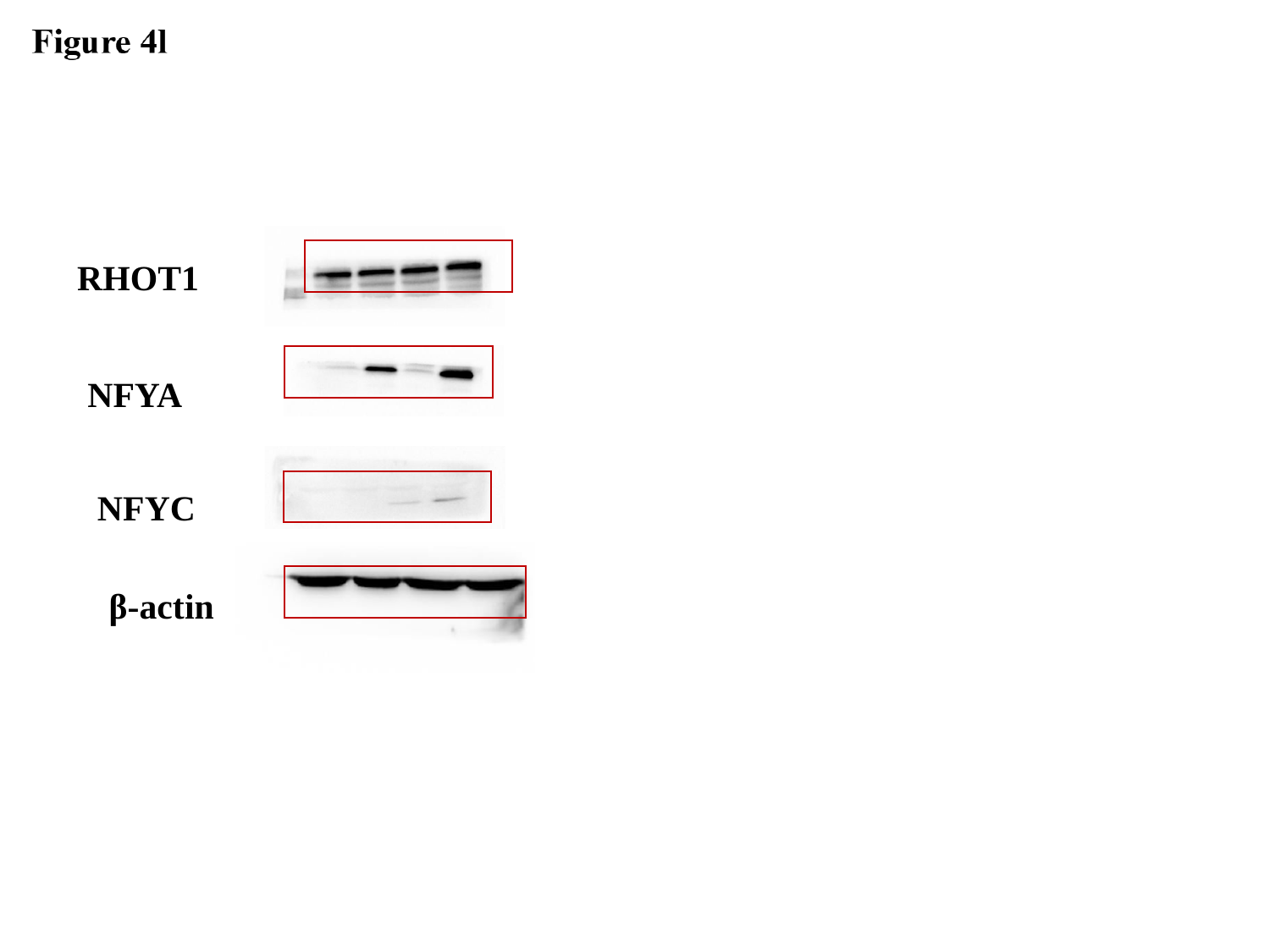

RHOT1
NFYA
NFYC
β-actin

## Slide 16
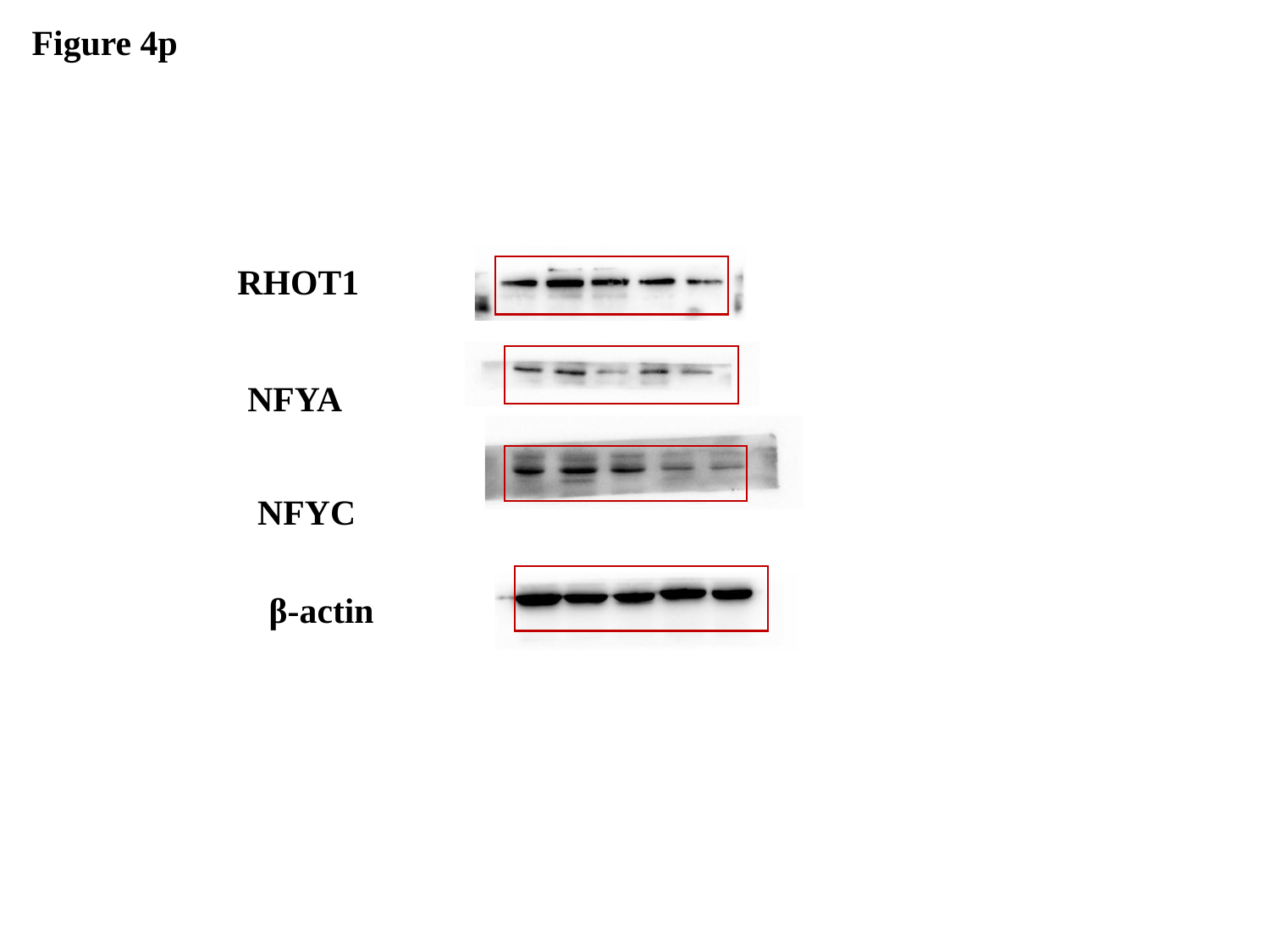

Figure 4p
RHOT1
NFYA
NFYC
β-actin

## Slide 17
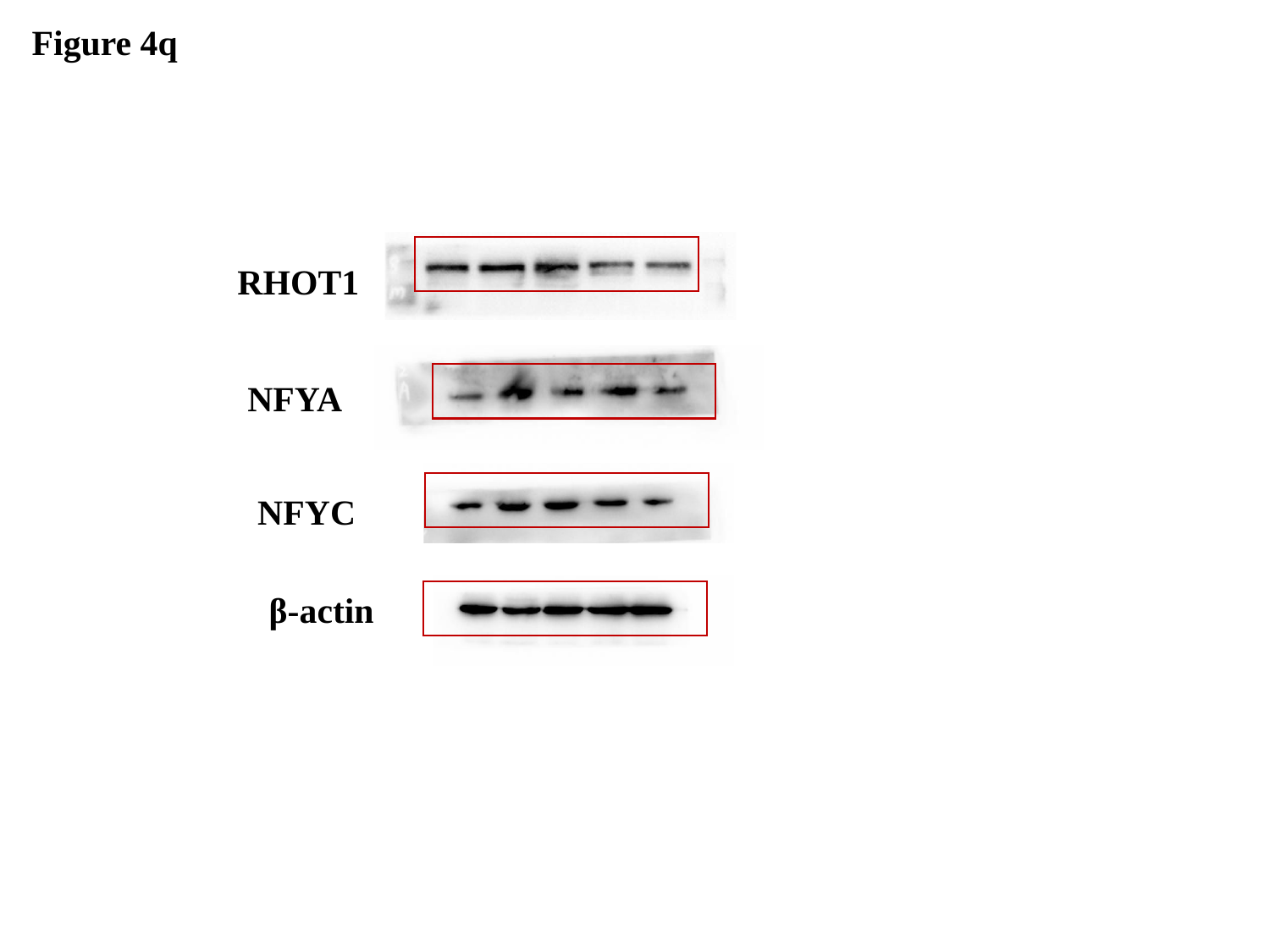

Figure 4q
RHOT1
NFYA
NFYC
β-actin

## Slide 18
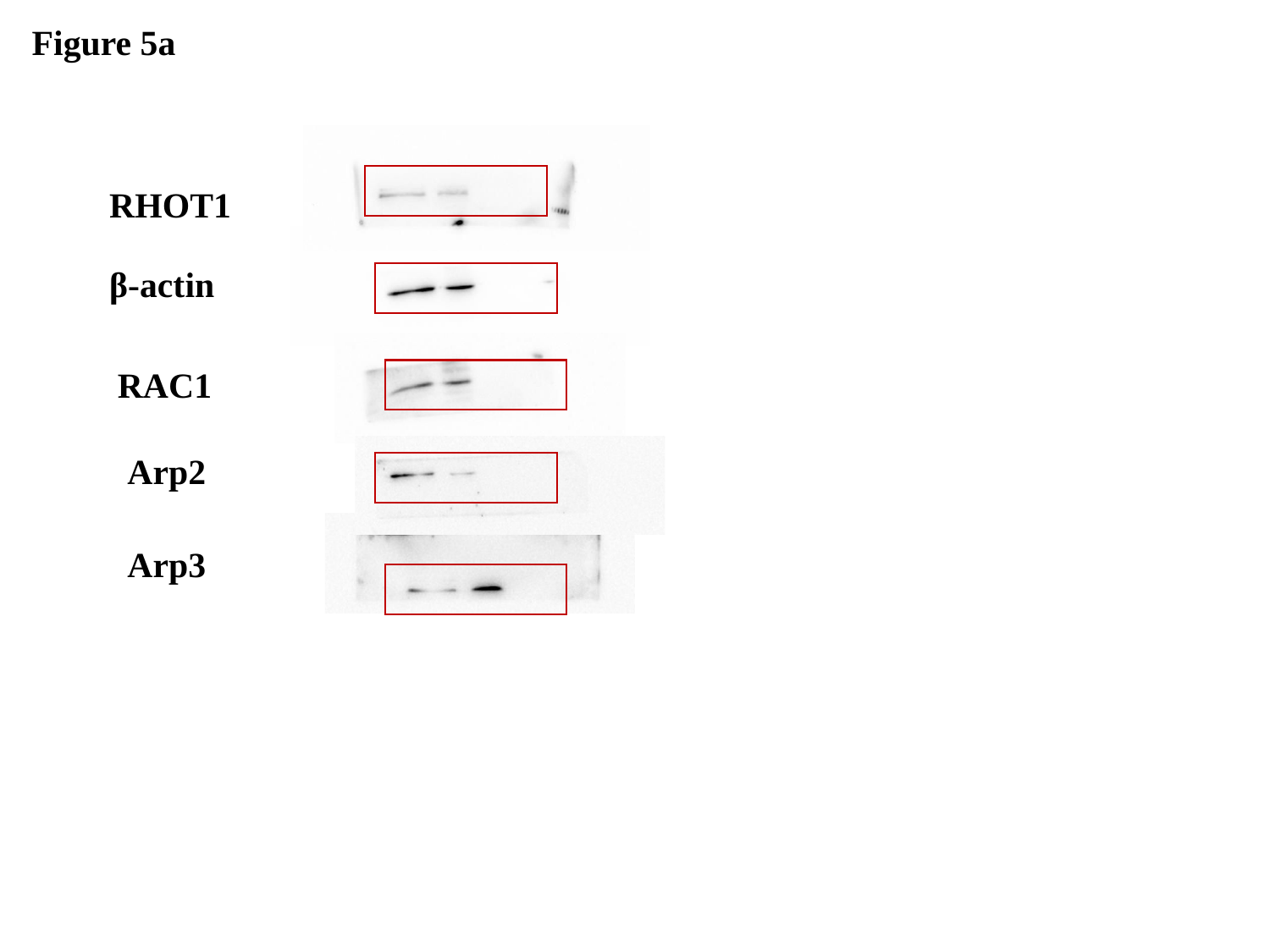

Figure 5a
RHOT1
β-actin
RAC1
Arp2
Arp3

## Slide 19
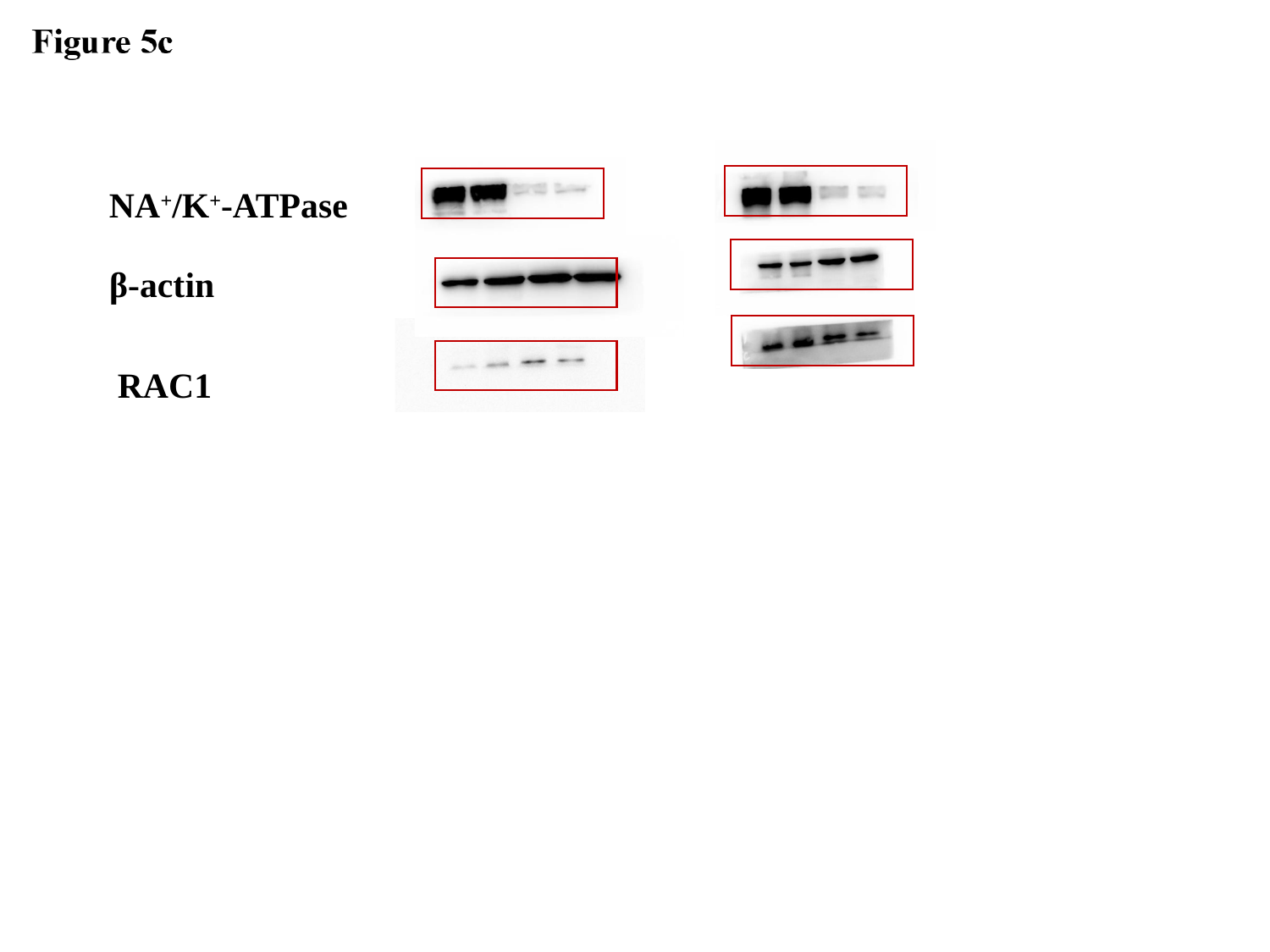

NA+/K+-ATPase
β-actin
RAC1

## Slide 20
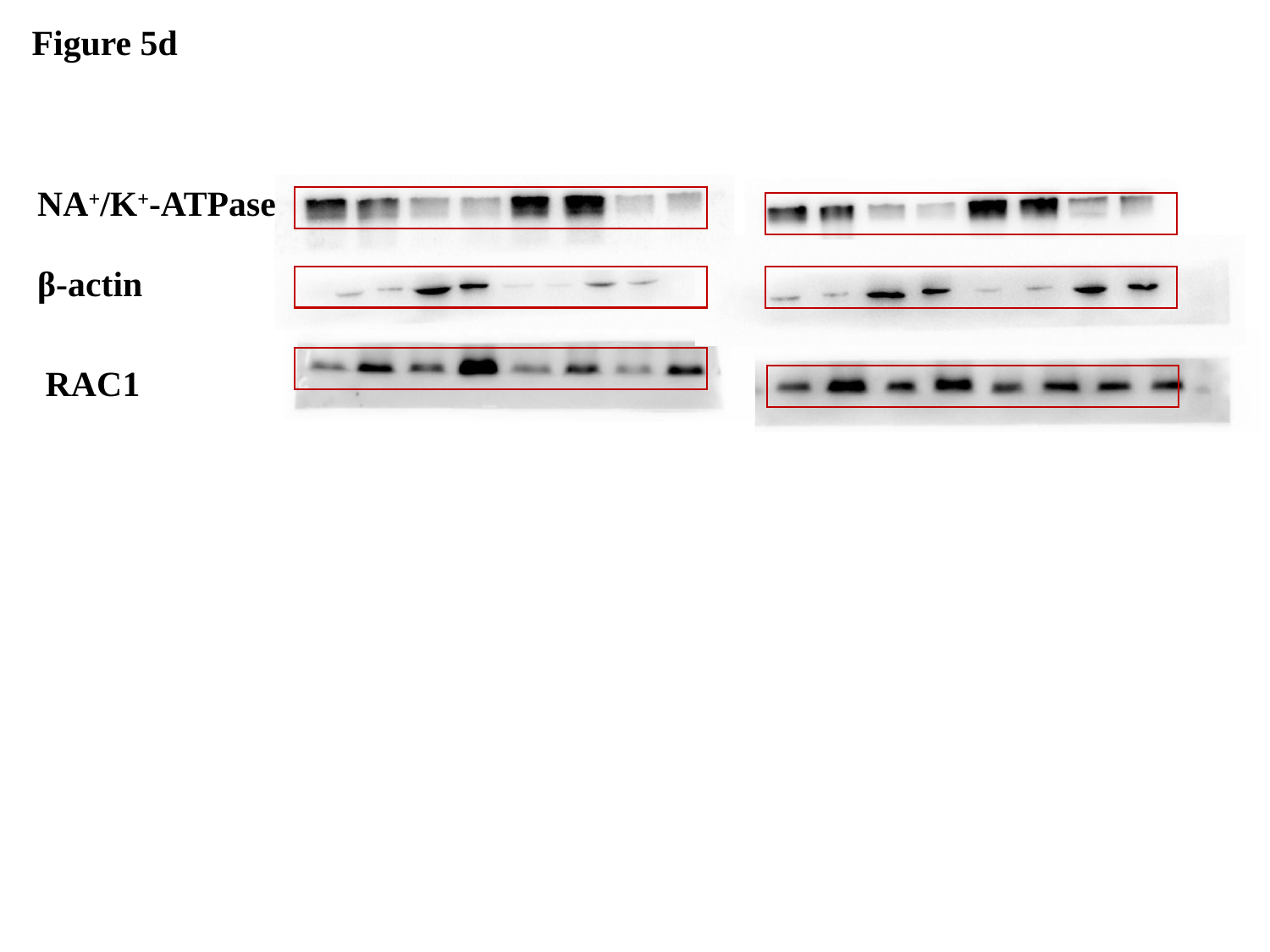

Figure 5d
NA+/K+-ATPase
β-actin
RAC1

## Slide 21
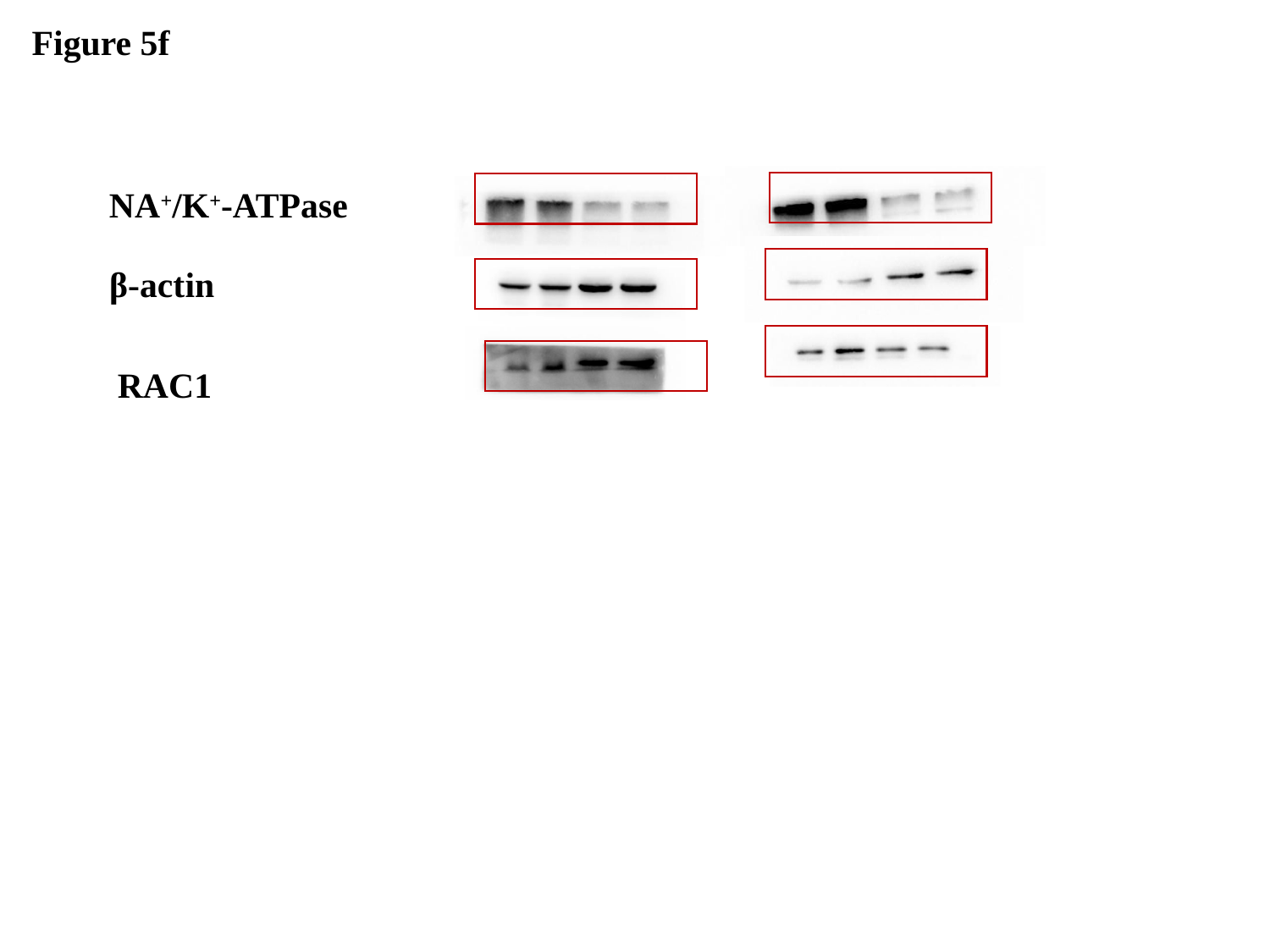

Figure 5f
NA+/K+-ATPase
β-actin
RAC1

## Slide 22
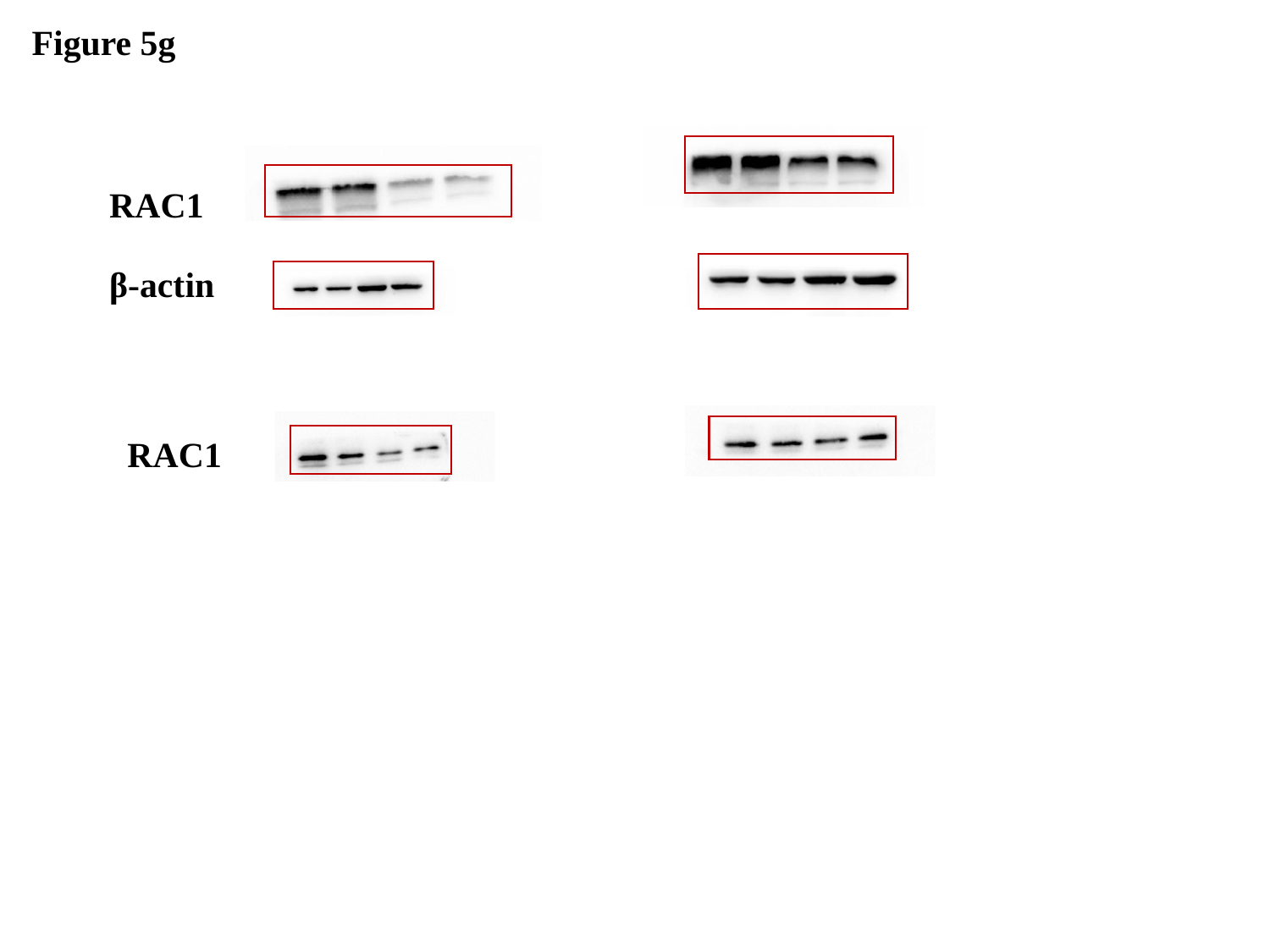

Figure 5g
RAC1
β-actin
RAC1

## Slide 23
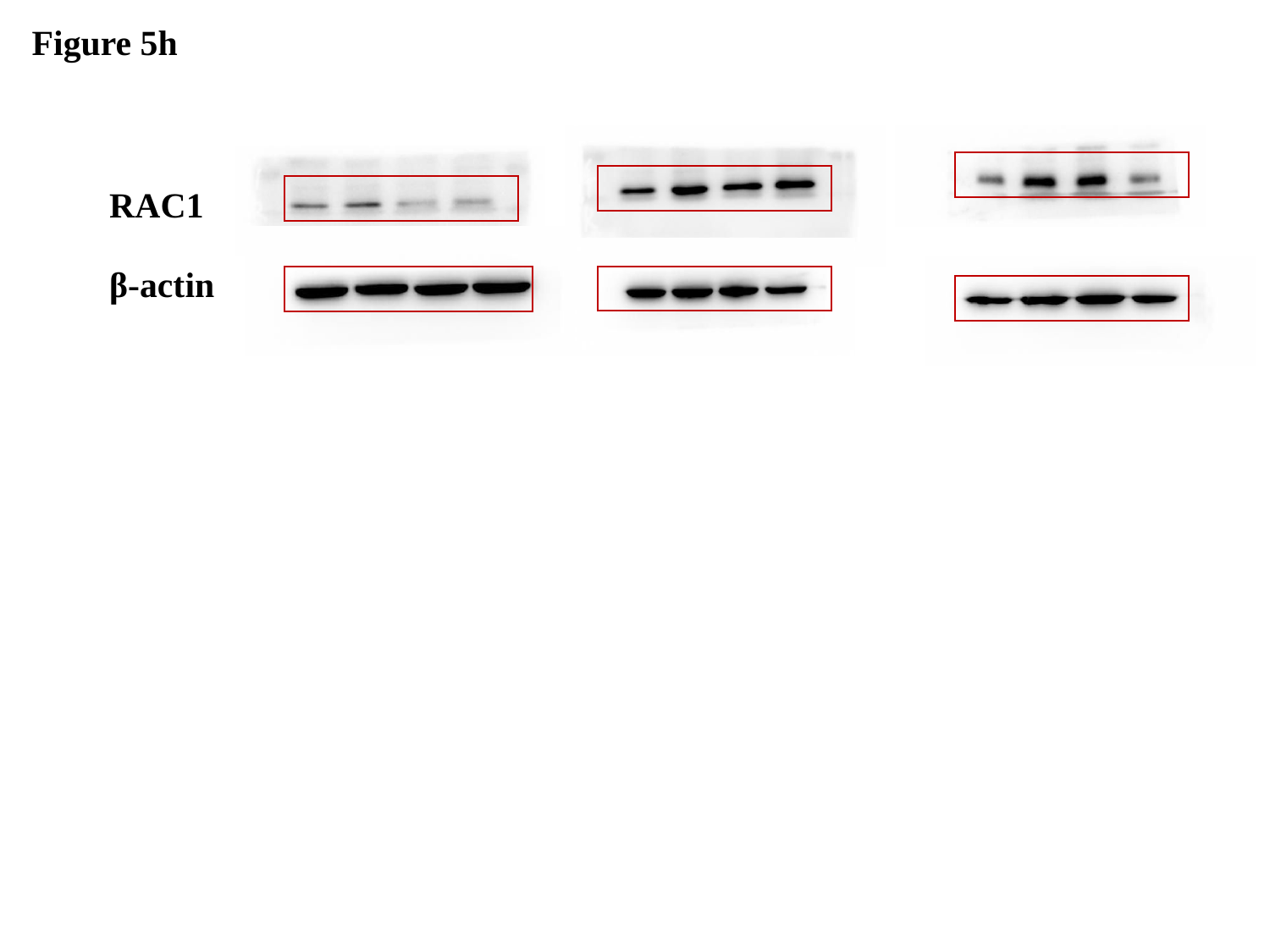

Figure 5h
RAC1
β-actin

## Slide 24
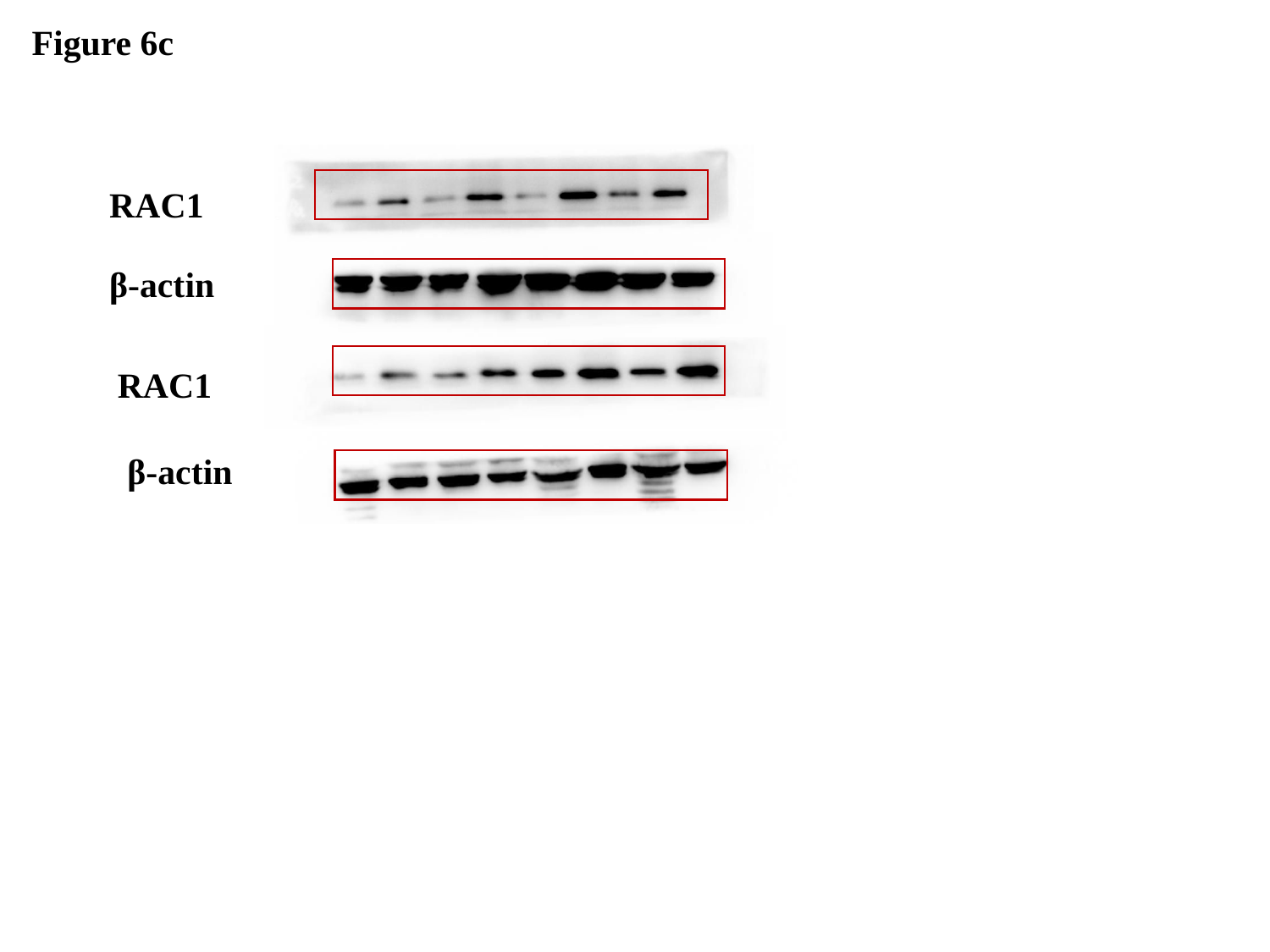

Figure 6c
RAC1
β-actin
RAC1
β-actin

## Slide 25
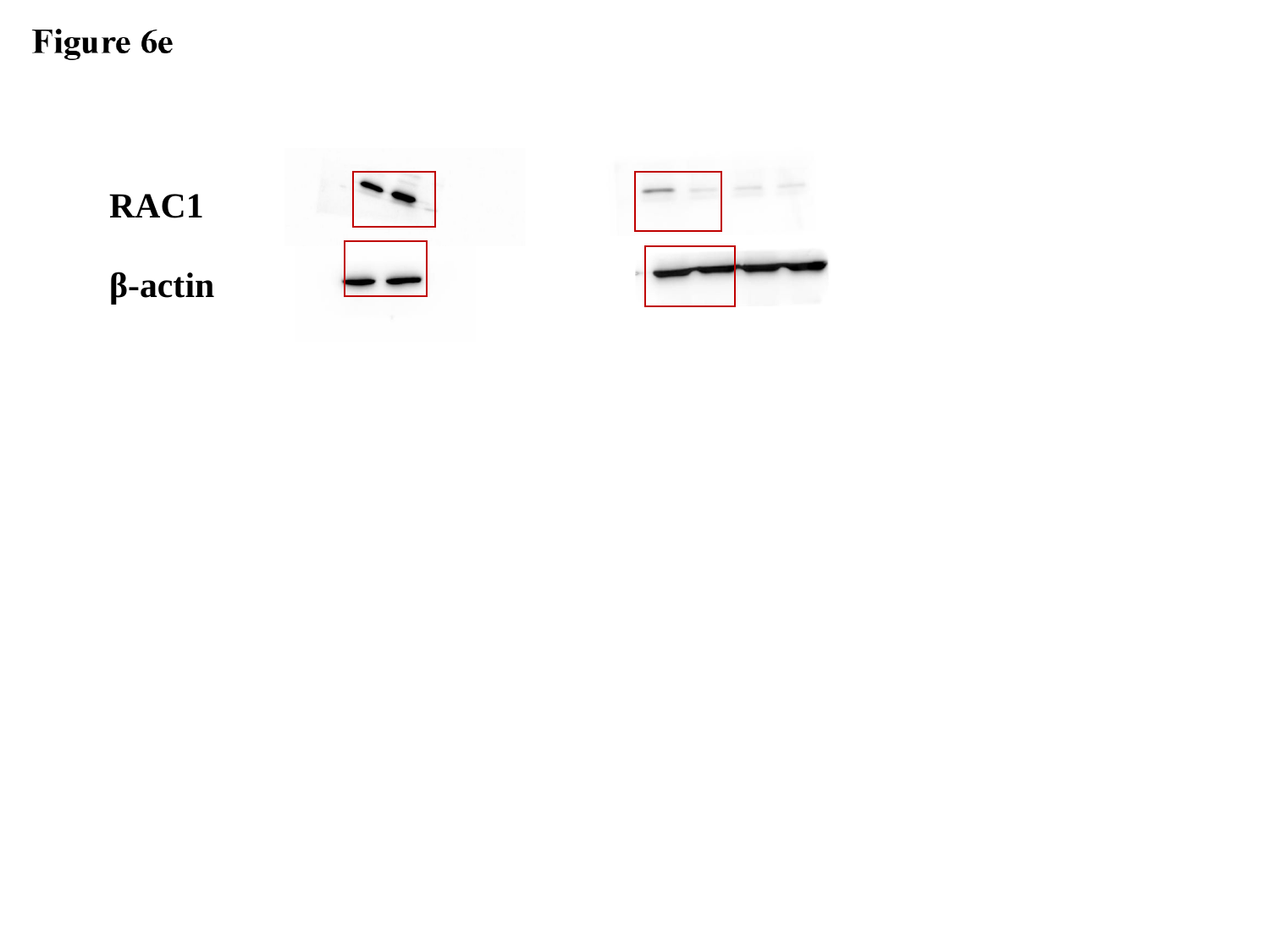

RAC1
β-actin

## Slide 26
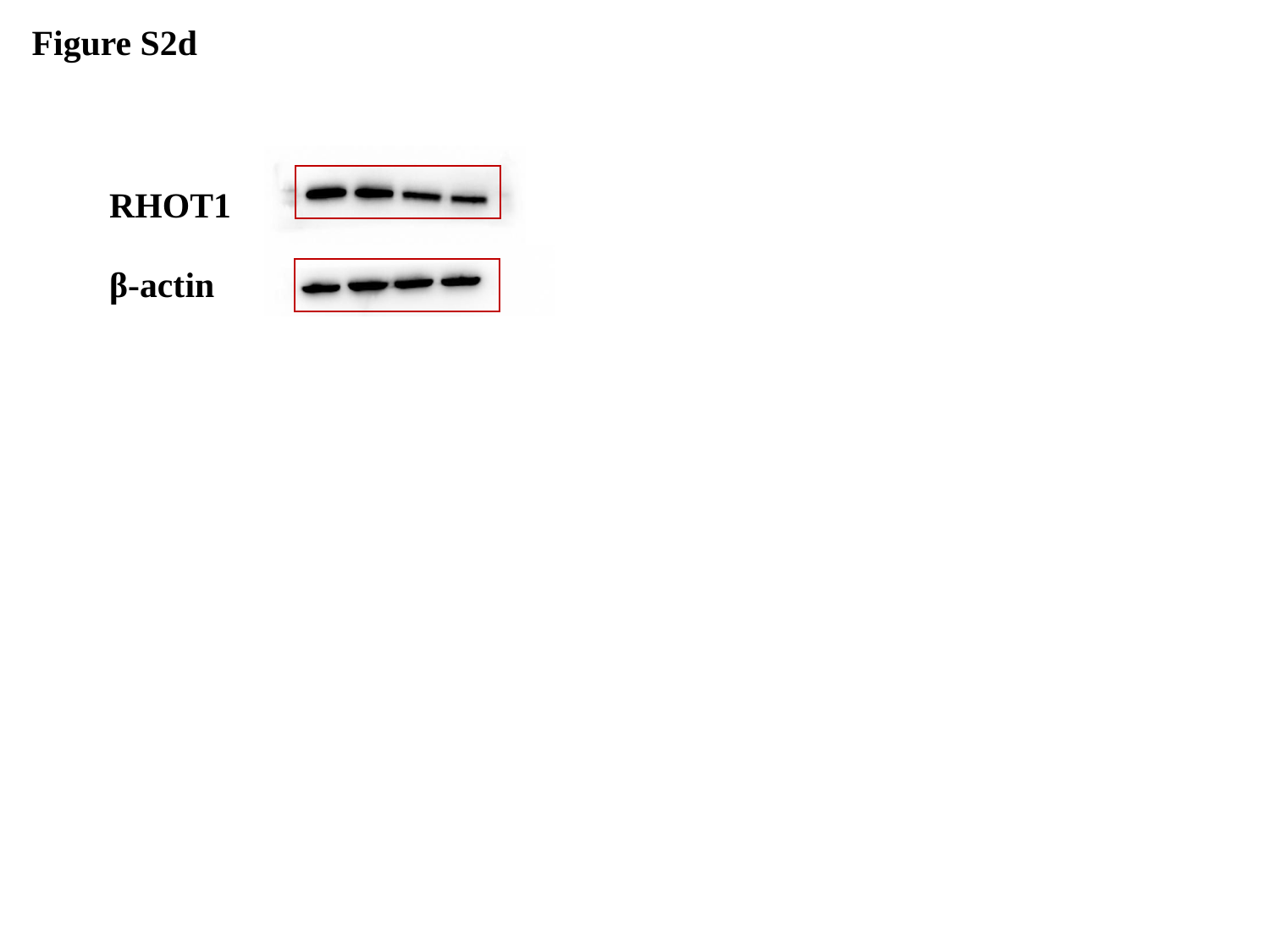

Figure S2d
RHOT1
β-actin
